# Gas Phase Thermal Reactions of *exo*-8-Cyclopropyl-bicyclo[4.2.0]oct-2-ene (1-*exo*)

**DOI:** 10.3390/molecules19021527

**Published:** 2014-01-27

**Authors:** Phyllis A. Leber, Anthony J. Nocket, William Hancock-Cerutti, Christopher Y. Bemis, Wint Khant Khine, Joseph A. Mohrbacher III, John E. Baldwin

**Affiliations:** 1 Department of Chemistry, Franklin & Marshall College, Lancaster, PA 17604-3003, USA; E-Mails: ajn5014@psu.edu (A.J.N.); william.hancock-cerutti@fandm.edu (W.H.-C.); christopher.bemis@fandm.edu (C.Y.B.); wintkhant.khine@fandm.edu (W.K.K.); joseph.mohrbacher@fandm.edu (J.A.M.); 2 Department of Chemistry, Syracuse University, Syracuse, NY 13244-4100, USA; E-Mail: jbaldwin@syr.edu

**Keywords:** gas phase reaction, thermal chemistry, [1,3]-sigmatropic rearrangement, [1,3]-carbon migration, CPC rearrangement

## Abstract

The title compound **1-*exo*** (with minor amounts of its C8 epimer **1-*endo***) was prepared by Wolff-Kishner reduction of the cycloadduct of 1,3-cyclohexadiene and cyclopropylketene. The [1,3]-migration product **2-*endo*** was synthesized by efficient selective cyclopropanation of *endo*-5-vinylbicyclo[2.2.2]oct-2-ene at the exocyclic π-bond. Gas phase thermal reactions of **1-*exo*** afforded C8 epimerization to **1-*endo***, [1,3]- migrations to **2-*exo*** and **2-*endo***, direct fragmentation to cyclohexadiene and vinylcyclopropane, and CPC rearrangement in the following relative kinetic order: *k_ep_* > *k_13 _* > *k_f_* > *k_CPC_*.

## 1. Introduction

Vinylcyclobutanes undergo ring expansion to cyclohexenes. Woodward and Hoffmann, in their *Conservation of Orbital Symmetry* treatise, formally classified this type of skeletal rearrangement as a [1,3]-sigmatropic carbon migration [1]. For a given vinylcyclobutane leading from a migrating carbon to a migration terminus, four discrete products could be formed by *si*, *sr*, *ai*, and *ar* routes. These designations refer to the potential for inversion (*i*) or retention (*r*) of configuration at the migrating carbon and suprafacial (*s*) or antarafacial (*a*) sigma bond formation at the migration terminus relative to the disposition of the original sigma bond with respect to the π bond framework. According to the Woodward-Hoffmann selection rules, the two symmetry-allowed products are *si* and *ar*, and the two symmetry-forbidden products are *sr* and *ai*. Given the geometric prohibition of antarafacial migration in bicyclic vinylcyclobutanes, only *si* and *sr* products can form without effecting excessive distortions of the molecular carbon skeleton. Although a one-step concerted process may have been assumed under orbital symmetry control of the vinylcyclobutane-to-cyclohexene rearrangement, recent experimental and computational studies converge toward a stepwise diradical mechanistic analysis [2].

Thermal reactions of bicyclo[3.2.0]hept-2-enes and bicyclo[4.2.0]oct-2-enes, despite their homologous relationship, afford different product stereoselectivities as well as preferred exit channels. The *si*/*sr* ratios for the more conformationally labile bicyclo[4.2.0]oct-2-enes are lower than those reported for bicyclo[3.2.0]hept-2-enes [3]. The relative kinetic importance of the observed exit channels for bicyclo[4.2.0]oct-2-enes labeled with a deuterium [4], methyl [5], or methoxy [3] at a migrating carbon is *k_ep_* > *k_f_* ≥ *k_13_*. The abbreviation *k_ep_* represents the rate of epimerization or one-centered stereomutation at C8; *k_f_*, the rate of direct fragmentation; *k_13_*, the total rate of [1,3]- sigmatropic migration including both *si* and *sr* products. This order is markedly different from the observation that [1,3]-carbon shifts afford the dominant products in the corresponding bicyclo[3.2.0]hept-2-enes [3,6,7]. An early review of [1,3]-carbon rearrangements offered a significant prediction of the role of exit channels such as fragmentation and epimerization in the mechanistic assessment process: “The nature of exit channels such as fragmentations and stereomutations, which are undoubtedly mediated by diradical transition structures, are important in the formulation of a consistent mechanistic framework” [2]. 

**Scheme 1:**
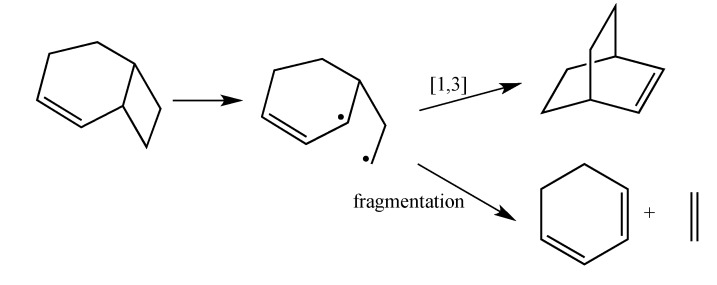
Gas Phase Reaction of Bicyclo[4.2.0]oct-2-ene.

**Scheme 2:**
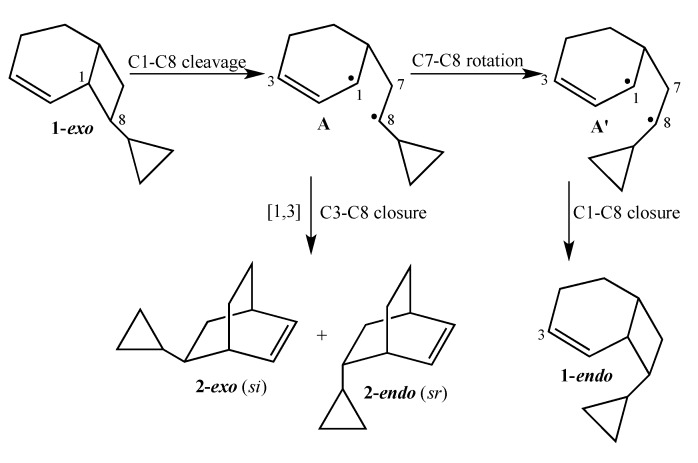
Gas Phase Reactions of **1-*exo***.

**Scheme 3:**
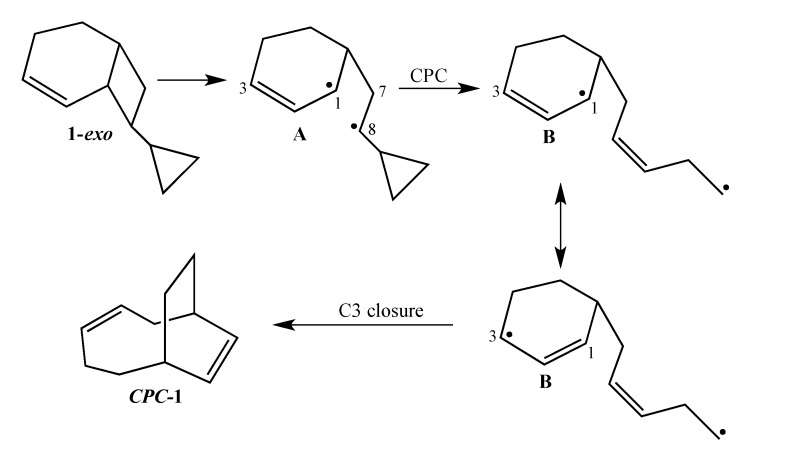
Potential CPC Ring Closure Product ***CPC*-1**.

The current mechanistic formulation for the vinylcyclobutane–to–cyclohexene rearrangement is a stepwise diradical process. Representations of this mechanism for the parent compound bicyclo[4.2.0]oct-2-ene are provided in Scheme 1, which shows that bicyclo[4.2.0]oct-2-ene can either isomerize via a [1,3]-shift to bicyclo[2.2.2]oct-2-ene or fragment to 1,3-cyclohexadiene and ethylene [4]. Due to the presence of a stereochemical marker at C8, the analog *exo*-8-cyclopropylbicyclo[4.2.0]oct-2-ene (**1-*exo***) can undergo [1,3]-sigmatropic migration to the *si* (**2-*exo***) and *sr* (**2-*endo***) products or C8 epimerization to **1-*endo*** (Scheme 2). The presence of a cyclopropyl substituent at C8 in **1-*exo*** affords a unique potential for cyclopropylcarbinyl (CPC)-to-homoallylic radical rearrangement of diradical **A** to diradical **B** (Scheme 3). Whereas key aspects of what transpires in a cyclopropylcarbinyl (CPC)-to-homoallylic radical rearrangement have been established for decades, this phenomenon has not yet been recognized in diradical species. Although no CPC rearrangement products were observed in the thermal reaction of *exo*-7-cyclopropylbicyclo[3.2.0]hept-2-ene, the argument that bicyclo[4.2.0]oct-2-enes might yield diradical transition structures with more “weakly interacting radical centers” [3] suggests the potential for **1-*exo*** to form a CPC product such as bicyclo[5.2.2]undeca-3,8-diene, ***CPC*-1** (Scheme 3).

## 2. Results and Discussion

### 2.1. Syntheses and Spectral Characterizations

**Scheme 4:**
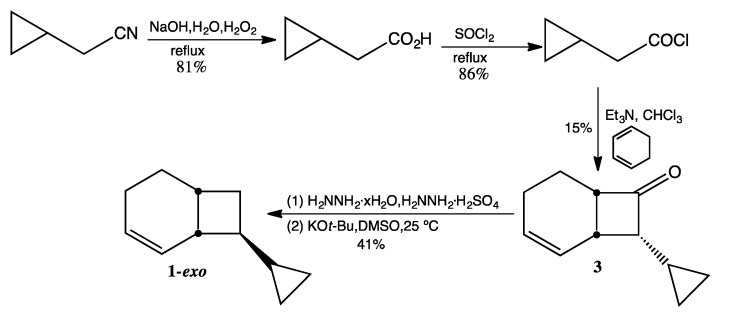
Synthesis of *exo*-8-Cyclopropylbicyclo[4.2.0]oct-2-ene (**1-*exo***).

Conversion of commercially available cyclopropylacetonitrile to cyclopropylacetyl chloride was accomplished by subjecting the nitrile to base-catalyzed hydrolysis [8], followed by reaction with thionyl chloride. Synthetic entry to the bicyclo[4.2.0]oct-2-ene skeleton of **1-*exo*** (Scheme 4) was achieved via ketene cycloaddition of 1,3-cyclohexadiene with cyclopropylketene, which was generated by treatment of cyclopropylacetyl chloride with triethylamine. A low-temperature Wolff-Kishner reduction subsequently converted the cyclobutanone hydrazone to a methylene moiety [9]. The basic conditions of the Wolff-Kishner reduction also resulted in epimerization at C8 to afford predominantly **1-*exo***. The GC retention time for the minor epimer **1-*endo***, which was present prior to purification, was diagnostic for identification of the **1-*endo*** product that resulted from C8 epimerization of **1-*exo***. Preparative GC subsequently produced **1-*exo*** in greater than 99% purity by GC analysis. 

The ^1^H- and ^13^C-NMR spectra of **1-*exo*** and its ketone precursor **3** appear distinctive due to the upfield signals for the cyclopropyl hydrogens and associated carbons. Whereas the ^1^H-NMR spectrum of **1-*exo*** exhibits three signals between 0.0 and 0.9 ppm for the five cyclopropyl hydrogens, the ketone **3** shows five unique signals between 0.1 and 0.8 ppm, each integrating for one hydrogen. While the ^13^C-NMR spectra of both **1-*exo*** and **3 ** have three shielded carbon signals, the cyclopropyl methine in ketone **3** appears considerably more upfield at 6.5 ppm relative to the corresponding methine in **1-*exo*** at 15.5 ppm due to the phenomenon of endo shielding [6,10].

**Scheme 5:**
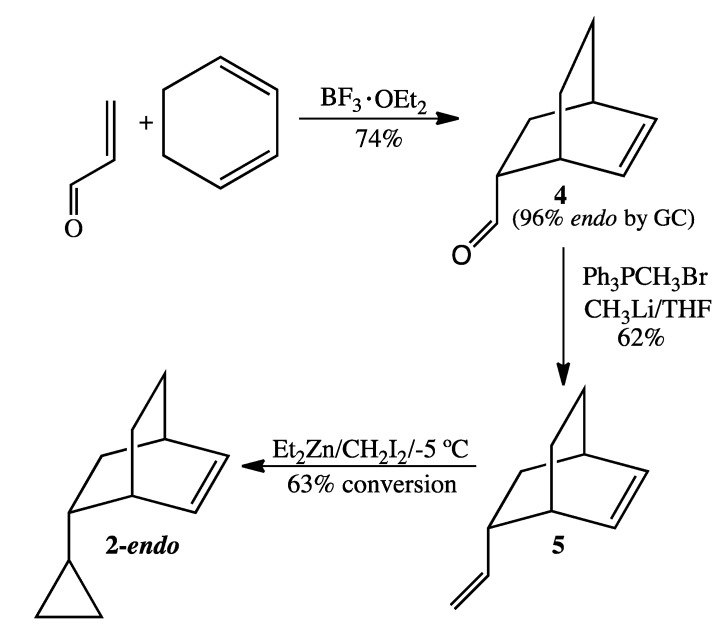
Synthesis of *endo*-5-Cyclopropylbicyclo[2.2.2]oct-2-ene (**2-*endo***).

Lewis acid-catalyzed Diels-Alder cycloaddition of 1,3-cyclohexadiene and acrolein with boron trifluoride yielded bicyclo[2.2.2]oct-5-en-2-carboxaldehyde (**4**) [5] almost exclusively as the endo epimer when the reaction was terminated between 2 and 4 h (Scheme 5). Wittig methylenation afforded 5-vinylbicyclo[2.2.2]oct-2-ene (**5**), whose ^13^C-NMR spectrum revealed four downfield and six upfield signals. In contrast to our failed attempt to secure 5-cyclopropylnorbornene by selective kinetic cyclopropanation of vinylnorbornene [11] using the Furukawa modification of the Simmons-Smith reaction [12], identical conditions resulted in excellent conversion of **5** to 5-cyclopropylbicyclo-[2.2.2]oct-2-ene (**2**). Our explanation for this high degree of regioselectivity is that the syn-hydrogens on the saturated -CH_2_CH_2_- bridge obstruct the exo face of the endocyclic olefin from reacting with the carbenoid complex. Similarly, the endo-vinyl group must also block the endo face of the endocyclic olefin. We thus observed relatively little endocyclic monocyclopropanation or dicyclopropanation, even when the reaction was allowed to proceed at room temperature. The reaction however ultimately reached a “steady state” when the ratio of **2-*endo***:**5** was 1.7:1. Preparative GC separation ultimately produced **2-*endo*** in greater than 98% purity by GC. 

The structure proof for **2-*endo*** relies heavily on NMR analysis. Five cyclopropyl hydrogens are observed between 0.0 and 0.5 ppm in the ^1^H-NMR spectrum, and the cyclopropyl carbons appear upfield of 20 ppm in the ^13^C-NMR spectrum. In addition, the ^13^C-NMR spectrum has two downfield signals between 132 and 135 ppm and six upfield signals between 24 and 45 ppm. Just as the methyl carbon in *endo*-5-methylbicyclo[2.2.2]oct-2-ene appears more downfield relative to the corresponding carbon in *exo*-5-methylbicyclo[2.2.2]oct-2-ene [5], the cyclopropyl methine in **2-*endo*** resonates at 18.1 ppm; in **2-*exo*** it resonates at 15.6 ppm. A sample of **2-*endo*** heated at 275 °C for 30 h did not undergo any observable thermal reaction. Due to this apparent thermal stability of compound **2** at 275 °C, ^13^C-NMR data for **2-*exo*** were obtained from a 120-h thermal reaction of **1-*exo*** exhibiting signals corresponding to both **2-*exo*** and **2-*endo***.

The identity of a CPC product eluting at 12.3 min, a retention time intermediate between that of **2-*exo*** (11.8 min) and **1-*exo*** (12.8 min), is suggestive of a bicyclic rather not a monocyclic structure. The potential bicyclic CPC product ***CPC*-1** identified in Scheme 3, or perhaps another CPC product, might yield distinctive mass spectral fragmentation patterns consistent with what was observed for the sole CPC product. The base peak at *m/z* 79 has been ascribed to the 1,3-cyclohexadienyl cation that forms from the cyclohexenyl radical cation by loss of a hydrogen atom. Similarly, the pentenyl radical cation at *m/z* 68 can undergo loss of a hydrogen atom to form the 1,3-pentadienyl cation. More minor fragments correspond to a C_7_H_10_ radical cation at *m/z* 94 due to extrusion of 1,3-butadiene from the molecular ion and to a C_9_H_12 _radical cation at *m/z* 120 due to loss of ethylene from the molecular ion.

**Scheme 6:**
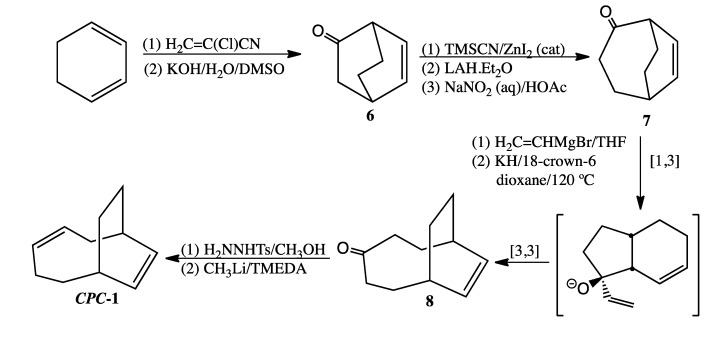
Synthesis of Potential CPC Product ***CPC*-1**.

Definitive characterization of ***CPC*-1** is based on independent synthesis using the synthetic route outlined in Scheme 6. Bicyclo[2.2.2]oct-5-en-2-one (**6**), a known compound, was prepared in high purity but low yield by Diels-Alder reaction of 1,3-cyclohexadiene and 2-chloroacrylonitrile followed by base-catalyzed hydrolysis [4,13]. Tiffaneau-Demjanov rearrangement to bicyclo[3.2.2]non-6-en-2-one (**7**) [14,15] was accomplished in 30% overall yield. Using the methodology of Uyehara [16], bicyclo[5.2.2]undec-8-en-4-one (**8**) was synthesized in 37% crude yield by treatment of compound **7** with vinylmagnesium bromide to effect transformation of the ketone moiety to a tertiary vinyl alcohol followed by a tandem sequence of alkoxide-promoted [1,3] sigmatropic rearrangement and Cope rearrangement. Conversion of **8** to ***CPC*-1** occurred through formation of the tosylhydrazone derivative of the ketone and subsequent Shapiro modification of the Bamford-Stevens reaction [17]. 

**Scheme 7:**
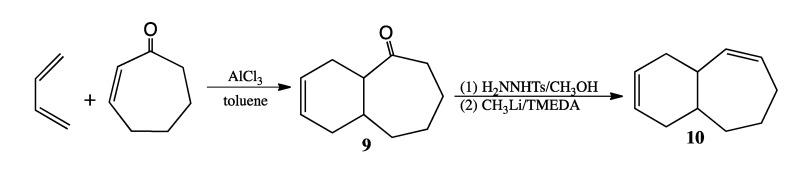
Synthesis of Bicyclo[5.4.0]undeca-2,9-diene (**10**).

An isomer of another potential CPC product, bicyclo[5.4.0]undeca-3,8-diene, was prepared using the synthetic methodology outlined in Scheme 7. Diels-Alder cycloaddition of 1,3-butadiene and cyclohept-2-enone in toluene using AlCl_3_ as a Lewis acid catalyst [18] proceeded in 47% yield. The resultant Diels-Alder cycloadduct bicyclo[5.4.0]undec-9-en-2-one (**9**) was obtained as a single product isomer after purification by column chromatography. Tosylhydrazone derivatization followed by the Shapiro modification of the Bamford-Stevens reaction resulted in bicyclo[5.4.0]undeca-2,9-diene (**10**) as the sole thermal product, which eluted at 16.2 min using a standard GC program and, unlike the CPC thermal product that eluted at 12.3 min, exhibited a prominent mass spec fragment at *m/z* 94 due to a cycloheptadienyl radical cation. We assumed, given the comparability in carbon framework between compound **10** and bicyclo[5.4.0]undeca-3,8-diene, that **10** and bicyclo[5.4.0]undeca-3,8-diene would have similar GC retention times and mass spectral fragmentation patterns. 

### 2.2. Thermal Reactions and Kinetic Analyses

**Figure 1:**
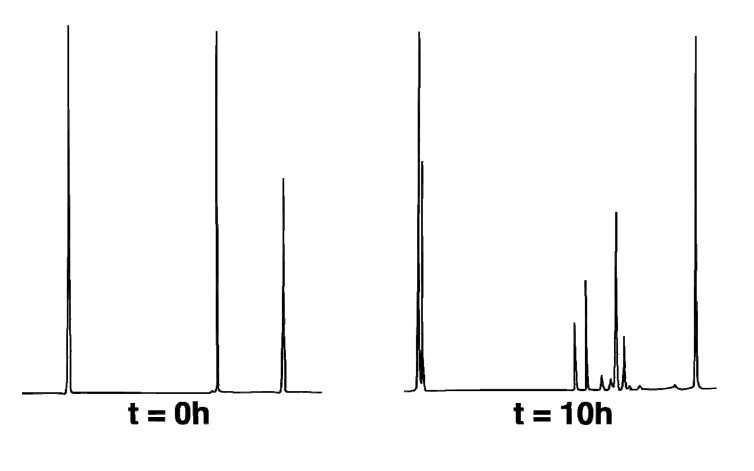
Capillary GC analysis of thermal reactions of **1-*exo*** @ 275 °C. At t = 0 h, the elution order is pentane < **1-*exo*** < ISTD. At t = 10 h, the elution order is pentane < 1,3-cyclohexadiene < **2-*endo*** < **2-*exo*** < ***CPC*-1** < **1-*exo*** < **1-*endo*** < ISTD.

**Scheme 8:**
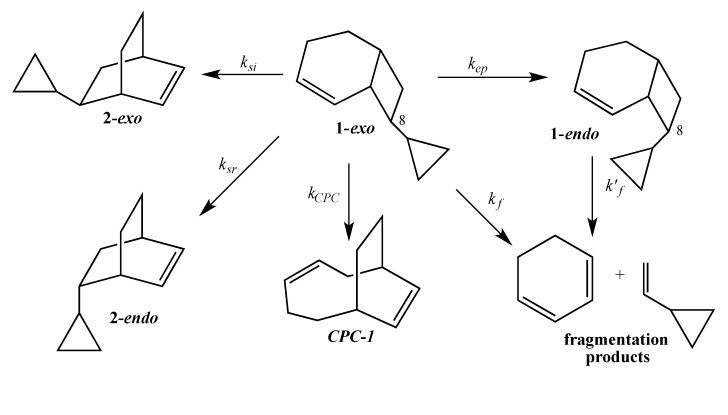
Gas Phase Thermal Profile of **1-*exo***.

The thermal reactions of **1-*exo***, as shown in Scheme 8, were followed at 275 °C in sealed base-treated capillary tubes that had been subjected to three freeze-pump-thaw cycles prior to closure. Capillary GC analysis provided relative concentration *versus* reaction time data for **1-*exo*** and for all isomeric products. As seen in Figure 1, all components were well-resolved and eluted in the following order: pentane < 1,3-cyclohexadiene < **2-*endo*** < **2-*exo*** < ***CPC*-1** < **1-*exo*** < **1-*endo*** < dodecane internal standard (ISTD). The value of the rate constant for overall loss of **1-*exo***, *k_0_* = 3.14 × 10^−5^ s^−1^, and its component rate constants were obtained using the Solver function in Microsoft Excel to fit experimental concentrations to the first-order exponential rate expressions based on the kinetic profile shown in Scheme 8. All other rate constants determined accordingly are as follows: *k*_si_ = 5.25 × 10^−6^ s^−1^, *k*_sr_ = 2.92 × 10^−6^ s^−1^, *k_ep_* = 1.9 × 10^−5^ s^−1^, k'_f_ = 7.3 × 10^−5^ s^−1^, and k_CPC_ = 6.5 × 10^−7^ s^−1^. The *si*/*sr* value thus derived from *k_si_* and *k_sr_* is 1.8, and *k_13_* = *k_si_* + *k_sr_* = 8.2 × 10^−6^ s^−1^. The rate of direct fragmentation *k_f_*, as determined by curve fitting, is 2.9 × 10^−6^ s^−1^. The difference between *k_0_* and the sum *k_13_* + *k_ep_* + k*_CPC_* approximated *k_f_* as 3.5 × 10^−6^ s^−1^. The relative order of importance of all kinetic processes is *k_ep_* > *k_13_* > *k_f_* > *k_ CPC_* (Table 1). 

**Table 1:** Exit Channels for *exo*-8-Substituted Bicyclo[4.2.0]oct-2-enes.

Entry #	Subst.	% epim	% frag	% [1,3]	*k_13_*/*k_f_*	*k_isom_*/*k_f_*	Rel. Rates	Ref. #
1	D-	67	22	11	0.5	3.5	*k_ep_* > *k_f_* > *k_13_*	4
2	CH_3_-	47	38	15	0.4	1.6	*k_ep_* > *k_f_* > *k_13_*	5
3	CH_3_O-	46	26	28	1.1	2.8	*k_ep_* > *k_f_* ~ *k_13_*	3
4 (**1-*exo***)	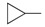	61	9	27	3.0	9.8	*k_ep_* > *k_13_* > *k_f_*	

According to Houk [19], “the dynamics of bond rotations on flat potential energy surfaces have significant influence on product distributions.” Due to the reversal in relative importance of [1,3]- rearrangement and fragmentation, the thermal profile of **1-*exo*** obviously differs from that of other bicyclo[4.2.0]oct-2-enes (Table 1). The exit channel data in Table 1 reveal a diminished contribution of fragmentation to the thermal manifold of **1-*exo*** rather than enhanced [1,3]-migrations. The resultant *k_13_*/*k_f_* ratio for **1-*exo*** (entry 4) is 3.0, the highest among the four entries in Table 1. This observation is consistent with our previous assertion [3] that “the *k_13_*/*k_f_* ratio might represent a qualitative measure of the inward migratory aptitude of the migrating carbon.” We attribute the low contribution from fragmentation to steric interaction, which could be alleviated when C8 undergoes an endo trajectory, between the exo-cyclopropyl substituent at C8 and the syn-hydrogens at the other three cyclobutane carbons. While the absolute contribution from [1,3]-carbon shifts is no greater for **1-*exo*** than for the *exo*-methoxy substrate (entry 3), the extent of epimerization is also greater for **1-*exo***. It should be noted that the isomerization process that affords the epimeric product is also a likely outcome of inward migration. A rough measure of inward *versus* outward migration for C8 can be obtained by dividing the sum of the rate constants for formation of isomeric products (*k_isom_* = *k_ep_* + *k_13_*) by the rate constant for formation of the fragmentation product (*k_f_*), as seen in the next to last column in Table 1. Based on this crude analysis, the inward:outward ratio for **1-*exo*** is ca. 10:1 for entry 4 compared to a value of ca. 3:1 for entry 3.

**Scheme 9:**
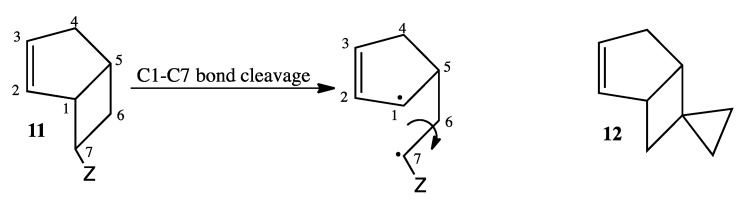
Structures of Compounds **11** and **12**.

Kinetic data reveal low stereoselectivity (Table 2, entry 3, column 5) and high reactivity (Table 3, entry 2, column 4) for reactant **1-*exo***. The *si*/*sr* value of 1.8 for **1-*exo*** is consistent with a longer-lived transition structure that can undergo more extensive rotation before the migrating carbon reaches the migration terminus and collapses to form the *si* or *sr* products. The same stereochemical trends are also apparent for the bicyclo[3.2.0]hept-2-enes (Table 2, column 4). Paradoxically, **1-*exo*** experiences relatively high reactivity, presumably due to conjugative stabilization of the rate-determining step transition state [11]. The potential stabilization that the cyclopropyl substituent offers the rate-determining transition structure might well prolong its lifetime, making **1-*exo*** anomalous with respect to the dependence of angular momentum on the mass of the substituent attached to C8. Carpenter has argued that C6-C7 bond torsion exerts the dominant influence on the “sense of rotation” of the migrating carbon C7 during [1,3]-carbon shifts in *exo*-7-substituted bicyclo[3.2.0]hept-2-enes (**11**), Scheme 9 [20]. In principle, substituents on C7 of greater mass should slow the C6-C7 rotation, thus affording greater stereoselectivity as determined by the *si*/*sr* ratio [3]. 

**Table 2:** Stereoselectivity of [1,3]-Shifts in Bicyclo[3.2.0]hept-2-enes and Bicyclo[4.2.0]oct-2-enes.

Entry #	Subst.	Mass	*si*/*sr* [3.2.0]	*si*/*sr* [4.2.0]	Ref. #
1	CH_3_O-	31	21	3.2	3
2	CH_3_-	15	7	2.4	5
3	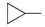	41	5	1.8 (**1-*exo***)	
4	D-	2	3	1.4	4

**Table 3:** Kinetic Data for Thermal Reactions of *exo*-8-Substituted Bicyclo[4.2.0]oct-2-enes @ 275 °C.

Entry #	Subst.	Subst Const σ_p_^+^	*k_0_* [4.2.0] (s^−1^)	log *k_0_* [4.2.0]	Ref. #
1	CH_3_O-	−0.65	4.3 × 10^−5^	−4.37	3
2 (**1-*exo***)	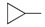	−0.48	3.1 × 10^−5^	−4.51	
3	CH_3_-	−0.31	1.5 × 10^−5^	−4.82	5
4	H-	0.00	1.7 × 10^−6^	−5.77	4

**Figure 2:**
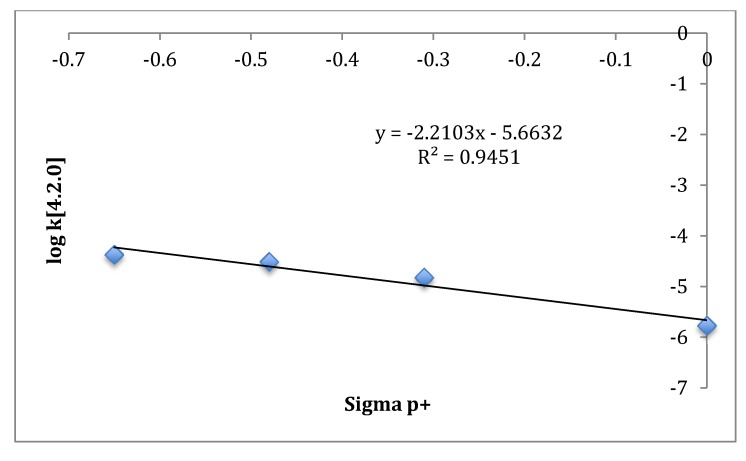
Hammett Plot for Bicyclo[4.2.0]oct-2-enes.

A linear free energy relationship analysis of exo-substituted bicyclo[4.2.0]oct-2-enes (Table 3 and Figure 2), as previously conducted for the exo-substituted bicyclo[3.2.0]hept-2-enes, also shows that the logarithm of the respective rate constants correlates well with the substituent constant σ_p_^+^, which possesses a large resonance contribution that can stabilize an electron-deficient radical center [11]. Although the negative slopes have comparable magnitudes, the presence of a minor contribution from a CPC product in the thermal profile of **1-*exo*** suggests that the radical centers in the purported transition structure are less closely associated, thus affording greater potential for a CPC-to-homoallylic radical rearrangement in **1-*exo*** compared to *exo*-7-cyclopropylbicyclo[3.2.0]hept-2-ene [11].

It is noteworthy that only one CPC product actually forms. A similar outcome was observed in the thermal chemistry of spiro[bicyclo[3.2.0]hept-2-ene-6,1'-cyclopropane] (**12**) in that only one of two potential CPC rearrangement products was observed [21]. The justification for this phenomenon was based on the assumption of a short lifetime for the resultant homoallylic radical that would preclude the exploration of all possible conformational space. We argued that the primary alkyl radical is sufficiently reactive that it will preferentially close at one end or the other of the allylic radical moiety depending on its proximity relative to the timing of the CPC rearrangement. A similar rationale might well account for the exclusive formation of ***CPC*-1** if the conversion of transition structure **A** to transition structure **B** (Scheme 3) occurs during an endo trajectory. If so, then the primary alkyl moiety of the homoallylic radical is significantly closer to C3 than to C1 of the allylic radical subunit of diradical **B**.

## 3. Experimental

### 3.1. General Information

Commercial reagents of high purity were purchased from Sigma-Aldrich (Milwaukee, WI, USA) and used without further purification. Unless otherwise indicated, all reactions were performed under an inert atmosphere of argon. Sigma-Aldrich silica gel, grade 923 (100–200 mesh), was used for flash column chromatography. NMR spectra were acquired on a Agilent INOVA 500 MHz instrument (Santa Clara, CA, USA). ^13^C-NMR hydrogen multiplicities for all compounds were obtained by a DEPT pulse sequence. All GC analyses were acquired on an HP cross-linked methyl silicone column (50 m × 0.2 mm i.d. × 0.10 µm film thickness). Preparative GC separations were accomplished on a GOW-MAC 580 GC.

### 3.2. Thermal Reactions

Thermal reactions of hydrocarbon **1-*exo*** were carried out at 275.0 °C (with temperature control to ± 0.1 °C provided by a Bayley Precision Temperature Controller Model 124) in based-treated capillary tubes immersed in a molten salt bath (composed of a eutectic mixture of NaNO_2_ and KNO_3_). Temperatures were measured with an Omega DP11 thermocouple with a digital readout to ± 0.1 °C. Run times were measured to ± 0.01 min with a Precision Solid State Time-it. The internal standard (ISTD) was dodecane. Thermolysis samples were analyzed on an HP 5890A GC equipped with an HP cross-lined methyl silicone column (50 m × 0.2 mm i.d. × 0.10 µm film thickness) operating at an initial temperature of 100 °C held for 1 min followed by a temperature ramp of 0.1 °C/min to a maximum temperature of 150 °C. Retention times (min) were as follows: 11.4 (**2-*endo***), 11.8 (**2-*exo***), 12.3 (***CPC*-1**), 12.8 (**1-*exo***), 13.0 (**1-*endo***), 15.5 (ISTD). 

### 3.3. Preparation of **1**-exo (Scheme 4)

*Cyclopropylacetic Acid*. The methodology of Fenick and Falvey [8] was employed for the hydrolysis of commercially available cyclopropylacetonitrile. ^1^H-NMR (500 MHz, *d*_6_-DMSO) δ 11.99 (br s, 1H), 2.09 (d, 2H), 0.92 (m, 1H), 0.44 (d, 2H), 0.11 (d, 2H). ^13^C-NMR (125 MHz, *d*_6_-DMSO) δ 174.2 (C=O), 38.9 (CH_2_), 7.0 (CH), 4.2 (2 CH_2_). FTIR (neat) ν_max _3007, 3010, 1704, 1222, 828 cm^−1^. 

*Cyclopropylacetyl chloride*. Cyclopropylacetic acid (10.0 g, 100 mmol) and thionyl chloride (14.6 mL, 200 mmol) were combined and refluxed overnight at 40 °C under argon. Short-path distillation at atmospheric pressure afforded two fractions: fraction 1 (bp ~75 °C, unreacted thionyl chloride) and fraction 2 (bp 130–135 °C, cyclopropylacetyl chloride, 10.2 g, 86%). ^1^H-NMR (500 MHz, CDCl_3_) δ 2.77 (d, 2H), 1.13 (m, 1H), 0.65 (d, 2H), 0.24 (d, 2H). ^13^C-NMR (125 MHz, CDCl_3_) δ 173.4 (C=O), 51.9 (CH_2_), 7.1 (CH), 4.6 (2 CH_2_). FTIR (neat) ν_max _3086, 3010, 1795, 1024, 925, 830, 707 cm^−1^. 

*endo-8-Cyclopropylbicyclo[4.2.0]oct-2-en-6-one* (**3**). Triethylamine (14.0 mL, 100 mmol), freshly distilled from CaH_2_, was dissolved in chloroform (60 mL, purified by washing with conc. H_2_SO_4_ and distilled from CaH_2_) and then added dropwise to a solution of cyclopropylacetyl chloride (12.0 g, 100 mmol) in 1,3-cyclohexadiene (80 mL, 850 mmol). After stirring at rt for 24 h, the chloroform was removed via simple distillation. After addition of 150 mL of ether, the suspended solid was removed by vacuum filtration. The ether layer was washed with water and then brine, dried over MgSO_4_ (anhydrous), and concentrated under reduced pressure. After removal of hydrocarbon fractions on a silica gel column with pentane as the eluting solvent, the ketone product was eluted with 95:5 pentane:ether. Removal of solvent by rotary evaporation from five fractions yielded relatively pure ketone (2.5 g, 15%). ^1^H-NMR (500 MHz, CDCl_3_) δ 5.95 (d, 2H), 3.49 (m, 1H), 3.01 (dt, 1H), 2.74 (dt, 1H), 2.04 (m, 2H), 1.99 (m, 1H), 1.52 (m, 1H), 0.77 (m, 1H), 0.61 (m, 1H), 0.42 (m, 1H), 0.29 (m, 1H), 0.14 (m, 1H). ^13^C-NMR (125 MHz, CDCl_3_) δ 212.4 (C=O), 129.6 (=CH), 126.3 (=CH), 66.5 (CH), 54.5 (CH), 27.7 (CH), 21.3 (CH_2_), 18.4 (CH_2_), 6.5 (CH), 4.5 (CH_2_), 2.2 (CH_2_). FTIR (neat) ν_max _3080, 3006, 1767, 1646, 685 cm^−1^. LRMS (EI) *m/z* 162 (M^+^, 1), 134 (5), 119 (5), 107 (8), 105 (10), 91 (32), 82 (27), 80 (100), 79 (61), 77 (25); HRMS (EI) calcd for C_11_H_14_O (M^+^) 162.1045, found 162.1043. 

*8-Cyclopropylbicyclo[4.2.0]oct-2-ene* (**1**). A mixture of hydrazine sulfate (2.1 g, 15.9 mmol) dissolved in hydrazine hydrate (10 mL) and compound **3 **(2.5 g, 15.4 mmol) was refluxed overnight at 65 °C. The reaction mixture was extracted several times with ether, and the combined organic layers were washed with distilled water and brine, dried over MgSO_4_ (anhydrous), and concentrated under reduced pressure to afford crude hydrazone (1.3 g, 48%). FTIR (neat) ν_max_ 3368, 3202, 3075, 3017, 1682, 1645, 1018, 739 cm^−1^. To a solution of potassium *tert*-butoxide (1.08 g, 9.0 mmol), sublimed under high vacuum at 190 °C and dissolved in 25 mL anhydrous DMSO, was added 8-cyclopropylbicyclo[4.2.0]oct-2-en-7-one hydrazone (1.3 g, 8.0 mmol) dropwise over 6 h. After stirring overnight, the reaction mixture was quenched with 5 mL of cold water and extracted four times with pentane. The combined pentane extracts were washed ten times with water to remove DMSO and dried over MgSO_4_ (anhydrous). The pentane was removed via simple distillation to yield crude product (1.02 g, 85%) in an exo:endo ratio of ca. 9:1. Preparative GC (on a 12' × ¼'' DC710 packed column at 130 °C) afforded **1-*exo*** in >99% purity by GC. ^1^H-NMR (500 MHz, CDCl_3_) δ 5.74 (m, 2H), 2.39 (br m, 2H), 1.99 (m, 1H), 1.93 (m, 1H), 1.70 (m, 2H), 1.64 (m, 2H), 1.48 (m, 1H), 0.87 (m, 1H), 0.37 (dd, 2H), 0.05 (dd, 2H). ^13^C-NMR (125 MHz, CDCl_3_) δ 130.3 (=CH), 127.0 (=CH), 46.2 (CH), 38.4 (CH), 29.2 (CH), 28.2 (CH_2_), 25.7 (CH_2_), 22.3 (CH_2_), 15.5 (CH), 2.9 (CH_2_), 2.8 (CH_2_). FTIR (neat) ν_max _3076, 3015, 1644, 1015, 697 cm^−1^. LRMS (EI) *m/z* 148 (M^+^, 1), 105 (3), 91 (8), 81 (9), 80 (100), 79 (45), 77 (11); HRMS (EI) calcd for C_11_H_16_ (M^+^) 148.1252, found 148.1255. 

### 3.4. Preparation of **2**-endo (Scheme 5)

*Bicyclo[2.2.2]oct-2-en-5-carboxaldehyde* (**4**). The catalyst BF_3_^.^OEt_2_ (2.0 mL, 16 mmol) was added under inert atmosphere to a solution of 1,3-cyclohexadiene (97%, 7.5 mL, 76 mmol) and acrolein (95%, 2.7 mL, 38 mmol) dissolved in anhydrous ether (50 mL). After stirring for 4 h, the reaction was quenched with ice-cold distilled water and then extracted with ether. The combined organic layers were washed sequentially with water, saturated sodium bicarbonate (aq), water, and brine and then dried over MgSO_4_. Concentration via rotary evaporation resulted in crude product (3.7 g, 71% yield), 96% of the endo epimer by GC analysis. ^1^H-NMR (500 MHz, CDCl_3_) δ 9.44 (d, 1H), 6.32 (t, 1H), 6.10 (t, 1H), 2.95 (br m, 1H), 2.63 (br m, 1H), 2.55 (br m, 1H), 1.72 (m, 1H), 1.65 (m, 2H), 1.55 (m, 1H), 1.35 (tt, 1H), 1.27 (m, 1H). ^13^C-NMR (125 MHz, CDCl_3_) δ 203.6 (O=CH), 136.0 (=CH), 130.6 (=CH), 50.8 (CH), 30.6 (CH), 29.2 (CH), 26.6 (CH_2_), 25.1 (CH_2_), 24.7 (CH_2_). FTIR (neat) ν_max _3044, 2937, 1721, 701 cm^−1^. LRMS (EI) *m/z* 136 (M^+^, C_9_H_12_O, 11), 108 (17), 93 (8), 91 (10), 80 (53), 79 (100).

*5-Vinylbicyclo[2.2.2]oct-2-ene* (**5**). A solution of recrystallized methyltriphenylphosphonium bromide (5.7 g, 16 mmol) in anhydrous THF (100 mL) was cooled to −78 °C and was treated slowly with 1.6 M CH_3_Li in ether (10.0 mL, 16 mmol). The resultant yellow suspension was then allowed to stir at 0 °C for 1 h. After again cooling the reaction mixture to −78 °C, bicyclo[2.2.2]oct-2-en-5-carboxaldehyde (2.0 g, 14.7 mmol) dissolved in anhydrous THF (5 mL) was added dropwise via syringe over 1 h. The reaction mixture, which was allowed to warm gradually to rt and to stir overnight, was then quenched with ice-cold water and extracted twice with pentane. The combined organic layers were washed with water and brine, dried over MgSO_4_, and concentrated by short-path simple distillation to yield crude diolefin product (1.5 g, 75%) as a yellow oil. The *endo*:*exo* ratio, as determined by GC analysis, was 96:4. ^1^H-NMR (500 MHz, CDCl_3_) δ 6.28 (t, 1H), 6.14 (t, 1H), 5.55 (pent, 1H), 4.86 (d, 1H), 4.79 (d, 1H), 2.46 (m, 2H), 1.75 (m, 1H), 1.55 (m, 1H), 1.46 (m, 1H), 1.24 (m, 3H), 1.06 (m, 1H). ^13^C-NMR (125 MHz, CDCl_3_) δ 145.4 (=CH), 134.8 (=CH), 132.0 (=CH), 111.6 (=CH_2_), 42.4 (CH), 35.7 (CH), 33.6 (CH_2_), 29.8 (CH), 26.1 (CH_2_), 24.4 (CH_2_). FTIR (neat) ν_max _3040, 2934, 1638, 996, 906, 693 cm^−1^. LRMS (EI) *m/z* 134 (M^+^, 8), 92 (8), 91 (15), 80 (100), 79 (76); HRMS (EI) calcd for C_10_H_14_ (M^+^) 134.1096, found 134.1093. 

*5-Cyclopropylbicyclo[2.2.2]oct-2-ene* (**2**). A solution of 1.0 M diethylzinc in hexanes (10.0 mL, 10.0 mmol) was added under argon to a dry round-bottomed flask at −40 °C. Sequential dropwise addition of 5-vinylbicyclo[2.2.2]oct-2-ene (1.0 g, 7.5 mmol) and diiodomethane (0.5 mL, 7.5 mmol) was performed. The reaction mixture was then allowed to warm to −5 °C and the temperature was held between −5 °C and 0 °C for 16 h, during which time additional aliquots of diethylzinc (4.0 mL) and diiodomethane (0.2 mL) were added to facilitate selective cyclopropanation of 5-vinylbicyclo-[2.2.2]0ct-2-ene at the exocyclic vinyl group. After 16 h, the reaction vessel was sealed with parafilm and stored overnight in a freezer at 0 °C. GC analysis the following day showed a 1.0:1.7 ratio of 5-vinylbicyclo[2.2.2]oct-2-ene to **2**, corresponding to a 63% conversion. The reaction was quenched with ice-cold water and extracted twice with pentane. The combined organic layers were washed with water and brine, dried over MgSO_4_, and concentrated by short-path simple distillation. The dominant epimer **2-*endo*** was obtained in >98% purity by prep GC (on a 12' × ¼'' DC710 packed column at 128 °C). ^1^H-NMR (500 MHz, CDCl_3_) δ 6.26 (dt, 1H), 6.20 (t, 1H), 2.49 (br m, 1H), 2.45 (br m, 1H), 1.72 (dq, 1H), 1.37 (m, 2H), 1.20 (m, 2H), 1.07 (m, 1H), 0.82 (m, 1H), 0.42 (m, 1H), 0.29 (m, 2H), 0.01 (m, 2H). ^13^C-NMR (125 MHz, CDCl_3_) δ 134.5 (=CH), 132.8 (=CH), 44.1 (CH), 35.3 (CH), 34.1 (CH_2_), 30.2 (CH), 26.4 (CH_2_), 24.6 (CH_2_), 18.1 (CH), 3.8 (CH_2_), 3.3 (CH_2_). FTIR (neat) ν_max _3075, 3043, 3000,1640, 703 cm^−1^. LRMS (EI) *m/z* 148 (M^+^, 1), 92 (4), 91 (7), 80 (100), 79 (35); HRMS (EI) calcd for C_11_H_16_ (M^+^) 148.1252, found 148.1253. 

A selective cyclopropanation of 5-vinylbicyclo[2.2.2]oct-2-ene to **2** at 20 °C resulted in 69% conversion. Selectivity for the desired product **2** was achieved regardless of temperature. The ratio of **2** to dicyclopropanated side product was 4.2:1 for the reaction at 20 °C compared to 5.0:1 for the reaction at 0 °C.

The thermal stability of **2*-endo*** was assessed by determining the **2*-endo***:dodecane (ISTD) ratio over a period of 30 h at 275 °C; sampling at 10 h increments afforded a fundamentally invariant ratio of 5.2 ± 0.2. 

A preparative 120-h thermal reaction of **1*-exo*** at 275 °C afforded a ca. 2:1 mixture of **2*-endo***:**2*-exo***. The LRMS of **2*-exo*** was virtually indistinguishable from that of **2*-endo***. The ^13^C-NMR signals due to **2*-exo*** were identified from the NMR spectrum of this epimeric mixture: δ 136.2 (=CH), 133.7 (=CH), 42.5 (CH), 34.8 (CH), 32.6 (CH_2_), 30.2 (CH), 26.3 (CH_2_), 20.1 (CH_2_), 15.6 (CH), 4.0 (CH_2_).

### 3.5. Preparation of CPC-**1** (Scheme 6)

*Bicyclo[2.2.2]oct-5-en-2-one* (**6**). Compound **6**, a low melting solid prone to sublimation, was prepared as previously reported [4]. ^1^H-NMR (500 MHz, CDCl_3_) δ 6.47 (t, 1H), 6.11 (t, 1H), 3.12 (br m, 1H), 2.97 (br m, 1H), 2.04 (s, 2H), 1.84 (m, 1H), 1.69 (m, 1H), 1.57 (m, 1H), 1.50 (m, 1H). ^13^C-NMR (125 MHz, CDCl_3_) δ 213.1 (C=O), 137.1 (=CH), 128.5 (=CH), 48.6 (CH), 40.5 (CH_2_), 32.4 (CH), 24.3 (CH_2_), 22.5 (CH_2_). FTIR (neat) ν_max _3051, 2945, 1717, 700 cm^−1^. LRMS (EI) *m/z* 122 (M^+^, 22), 80 (100), 79 (81); HRMS (EI) calcd for C_8_H_10_O (M^+^) 122.0732, found 122.0729. 

*Bicyclo[3.2.2]non-6-en-2-one* (**7**). Subjecting 1.12 g (9.18 mmol) of **6** to Tiffaneau-Demjanov ring expansion [14] afforded 0.36 g (29% yield) of **7** (contaminated with 7% bicyclo[3.2.2]non-6-en-3-one) after purification by column chromatography. ^1^H-NMR (500 MHz, CDCl_3_) δ 6.35 (t, 1H), 6.08 (t, 1H), 3.05 (br t, 1H), 2.69 (br m, 1H), 2.56 (t, 2H), 1.97 (m, 1H), 1.89 (m, 1H), 1.82 (m, 4H). ^13^C-NMR (125 MHz, CDCl_3_) δ 209.9 (C=O), 136.7 (=CH), 127.9 (=CH), 49.3 (CH), 39.4 (CH_2_), 31.2 (CH), 30.7 (CH_2_), 24.9 (CH_2_), 24.1 (CH_2_). FTIR (neat) ν_max _3039, 2940, 1693, 705 cm^−1^. LRMS (EI) *m/z* 136 (M^+^, 20), 118 (50), 117 (38), 92 (85), 80 (57), 79 (100). 

*Bicyclo[5.2.2]undec-8-en-4-one* (**8**). A sample of 0.34 g (2.5 mmol) **7** was allowed to react gradually with 4.0 mL (4.0 mmol) of 1.0 M vinylmagnesium bromide in THF under argon at −65 °C. Workup by treatment with 1 M NH_4_Cl (aq) followed by successive extraction with ether and subsequent drying over MgSO_4_ afforded 0.32 g (2.0 mmol, 78%) of crude vinyl alcohol as a pair of diastereomers. FTIR (neat) ν_max _3424, 3032, 2975, 1631, 998, 916, 720 cm^−1^. LRMS (EI) *m/z* 164 (M^+^, 2), 146 (14), 131 (17), 104 (64), 92 (81), 91 (71), 83 (100), 79 (100). The vinyl alcohols were treated with 0.29 g (2.1 mmol) of KH (30 wt% in mineral oil) and 18-crown-6 (1.09 g, 4.1 mmol) dissolved in 20 mL anhydrous dioxane under reflux at 120 °C for 1.5 h. The major product in the crude mixture (0.16 g, 47%) corresponded to **8**, as confirmed by ^13^C-NMR spectral comparison with reported literature values [16]. ^13^C-NMR (125 MHz, CDCl_3_) δ 219.0 (C=O), 133.1 (=CH), 41.0 (CH_2_), 31.7 (CH_2_), 30.5 (CH), 25.4 (CH_2_). FTIR (neat) ν_max_ 3019, 2918, 1697, 702 cm^−1^. LRMS (EI) *m/z* 164 (M^+^, 23), 136 (75), 118 (82), 117 (50), 92 (100), 91 (69), 79 (100). 

*Bicyclo[5.2.2]undeca-3,8-diene* (***CPC*-1**). To a solution of 150 mg (0.81 mmol) of *p*-toluenesulfonyl hydrazide in 1 mL of methanol was added crude **8** (150 mg, 0.92 mmol). The resultant crystals were filtered and washed with cold 1:1 pentane-ether (1 mL). The tosylhydrazone derivative of **8** (0.02 g, 0.09 mmol) was then suspended in TMEDA (anhydrous, 0.2 mL). After cooling the mixture in a dry ice/acetone bath, 1.6 M CH_3_Li in diethyl ether (4.4 equiv., 0.25 mL, 0.4 mmol) was added dropwise via syringe. The resultant solution turned dark orange in color. After stirring overnight, the reaction was quenched with 1:1 pentane-water, washed successively with 2 M HCl (aq) and 2M NaOH (aq), and then dried over MgSO_4_. Removal of solvent by short-path distillation gave a trace amount of liquid. Major component LRMS (EI) *m/z* 148 (M^+^, 11), 133 (13), 120 (29), 106 (29), 93 (30), 92 (36), 91 (50), 79 (100). This mass spectrum is virtually identical to that of the thermal product eluting at 12.3 min (see SI).

### 3.6. Preparation of Bicyclo[5.4.0]undeca-2,9-diene (Scheme 7)

*Bicyclo[5.4.0]undec-9-en-2-one* (**9**). A sample of 1.63 g (12.2 mmol) of AlCl_3_ was weighed into a thick-walled flask under argon in a glovebag. A solution of 2-cycloheptenone (1.5 mL, 0.988 g/mL, 13.6 mmol) in anhydrous toluene (7.6 mL) was transferred to the flask via syringe. After complexation between the ketone and Lewis acid had proceeded for 40 min at rt, a solution of 1,3-butadiene in toluene (27.4 mL, 20 wt%, 0.806 g/mL, 81.7 mmol) was added via syringe through a septum, which was then replaced with Teflon screw cap. After stirring the reaction at rt for 22 h, the reaction mixture was extracted with ether, and the organic layer was washed twice with water and once with brine. Elution of the ketone from a silica column with 98:2 pentane:ether afforded 1.04 g (6.4 mmol, 47%) of pure ketone **9**. ^1^H-NMR (500 MHz, CDCl_3_) δ 5.68 (m, 1H), 5.62 (m, 1H), 2.76 (dt, 1H), 2.65 (m, 1H), 2.45 (dd, 1H), 2.29 (br m, 3H), 2.17 (m, 1H), 1.89 (m, 1H), 1.80 (m, 2H), 1.66 (m, 4H). ^13^C-NMR (125 MHz, CDCl_3_) δ 215.2 (C=O), 125.4 (=CH), 125.3 (=CH), 49.7 (CH), 43.7 (CH_2_), 34.0 (CH), 33.3 (CH_2_), 31.7 (CH_2_), 27.5 (CH_2_), 26.2 (CH_2_), 24.1 (CH_2_). FTIR (neat) ν_max _3023, 2921, 1691, 1654, 653 cm^−1^. LRMS (EI) *m/z* 164 (M^+^, 45), 146 (29), 135 (27), 117 (46), 104 (55), 91 (49), 79 (100), 77 (47). 

*Bicyclo[5.4.0]undeca-2,9-diene* (**10**). Ketone **9** (0.52 g, 3.2 mmol) was added to a solution of *p*-toluenesulfonyl hydrazide (0.45 g, 2.4 mmol) in methanol (4 mL). After sitting overnight, a crop of crystals (0.75 g, 68%) was obtained. The tosylhydrazone of ketone **9** was dried in a vacuum oven: mp 137–139 °C. Dropwise addition of a solution of 1.6 M CH_3_Li in pentane (4.0 mL, 6.4 mmol) at −55 °C over 15 min to a suspension of the tosylhydrazone (0.60 g, 1.7 mmol) suspended in 4 mL anhydrous TMEDA produced a dark red solution. After stirring overnight at rt, the reaction was quenched with cold water and extracted with pentane. Removal of the solvent by short-path distillation yielded product **10** (0.13 g, 0.88 mmol, 52%), which was purified via prep GC (on a 12' × ¼'' DC710 packed column at 130 °C) for purposes of spectral analysis. ^1^H-NMR (500 MHz, CDCl_3_) δ 5.79 (m, 1H), 5.66 (m, 2H), 5.44 (dd, 1H), 2.68 (br s, 1H), 2.24 (br d, 1H), 2.11 (m, 3H), 1.99 (br d, 1H), 1.88 (m, 2H), 1.85 (m, 1H), 1.72 (m, 1H), 1.54 (m, 1H), 1.48 (m, 1H). ^13^C-NMR (125 MHz, CDCl_3_) δ 135.8 (=CH), 132.0 (=CH), 127.2 (=CH), 125.5 (=CH), 37.5 (CH), 35.3 (CH_2_), 34.6 (CH), 32.5 (CH_2_), 29.1 (CH_2_), 28.9 (CH_2_), 23.4 (CH_2_). LRMS (EI) *m/z* 148 (M^+^, 41), 133 (13), 119 (14), 105(21), 94 (84), 92 (31), 91 (48), 79 (100), 77 (25). 

## 4. Conclusions

The low stereoselectivity (*si*/*sr* = 1.8) observed for **1-*exo*** is consistent with a longer lifetime for diradical transition structure **A** (Scheme 3) with more opportunities for rotation about the C7-C8 bond. The high reactivity observed for **1-*exo*** can be accounted for by cyclopropyl conjugative stabilization of transition structure **A** (Scheme 2). As confirmation of the proposed electronic stabilization, a Hammett plot shows that the logarithm of the respective rate constants for a series of 8-substituted bicyclo[4.2.0]oct-2-enes correlates with the substituent constant σ_p_^+^, which possesses a large resonance contribution. Finally, formation of only one CPC product (***CPC*-1) **suggests a short lifetime for diradical transition structure **B** (Scheme 3) that has insufficient time to explore all of conformational space and thus is unable to access all ring closure exit channels. ***CPC*-1** is realized before intramolecular reorganization of the side chain results in other potential CPC products. 

## Supplementary Materials

The NMR spectra of compounds **1-*exo***, **2-*endo***, **8**, and **10** and relevant kinetic data for thermal reactions of **1-*exo*** can be accessed at: http://www.mdpi.com/1420-3049/19/2/1527/s1.

## References

[1] Woodward R.B., Hoffmann R. (1970). The Conservation of Orbital Symmetry.

[2] Leber P.A., Baldwin J.E. (2002). Thermal [1,3] Carbon Sigmatropic rearrangements of vinylcyclobutanes. Acc. Chem. Res..

[3] Leber P.A., Lasota C.C., Strotman N.A., Yen G.S. (2007). The effect of a methoxy substituent on the vinylcyclobutane carbon migration. J. Org. Chem..

[4] Powers D.C., Leber P.A., Gallagher S.S., Higgs A.T., McCullough L.A., Baldwin J.E. (2007). Thermal chemistry of bicyclo[4.2.0]oct-2-enes. J. Org. Chem..

[5] Bogle X.S., Leber P.A., McCullough L.A., Powers D.C. (2005). Thermal reactions of 8-methylbicyclo[4.2.0]oct-2-enes: Competitive diradical-mediated [1,3] sigmatropic, stereomutation, and fragmentation processes. J. Org. Chem..

[6] Bender J.D., Leber P.A., Lirio R.R., Smith R.S. (2000). Thermal rearrangement of 7-methylbicyclo[3.2.0]hept-2-ene: An experimental probe of the extent of orbital symmetry control in the [1,3] sigmatropic rearrangement. J. Org. Chem..

[7] Baldwin J.E., Leber P.A. (2001). New thermal reactions of deuterium-labeled bicyclo[3.2.0]hept-2-enes: Bicyclic skeletal inversion and epimerization at C7. J. Am. Chem. Soc..

[8] Fenick D.J., Falvey D.E. (1994). Free Radical Rearrangements in Uracil Derivatives. J. Org. Chem..

[9] Burkey J.D., Leber P.A., Silverman L.S.  (1986). Preparation of 7-methyl-7-vinylbicyclo[3.2.0]hept-2-ene by cyclobutanone reduction. Synth. Commun..

[10] Rey M., Roberts S., Dieffenbacher A., Dreiding A.S. (1970). Stereochemische aspekte der addition von ketenen an cyclopentadien. Helv. Chim. Acta.

[11] Leber P.A., Nocket A.J., Wipperman M.F., Zohrabian S., Bemis C.Y., Sidhu M.K. (2013). Experimental evidence of a cyclopropylcarbinyl conjugative electronic stabilization effect. Org. Biomol. Chem..

[12] Furukawa J., Kawabata N., Nishimura J. (1968). Synthesis of cyclopropanes by the reaction of olefins with dialkylzinc and methylene iodide. Tetrahedron.

[13] Carrupt P.-A., Vogel P. (1989). The carbonyl group as homoconjugated electron-releasing substituent. Regioselective electrophilic additions at bicyclo[2.2.1]hept-5-en-2-one, bicyclo[2.2.2]oct-5-en-2-one, and derivatives. Helv. Chim. Acta.

[14] Uyehara T., Kabasawa Y., Furuta T., Kato T. (1986). Rearrangement approaches to cyclic skeletons. III. Practical route to *cis*-bicyclo[4.3.0]non-4-en-7-ones based on photochemical [1,3] acyl migration of bicyclo[3.2.2]non-6-en-2-ones. Bull. Chem. Soc. Jpn..

[15] Patel V., Ragauskas A.J., Stothers J.B. (1986). ^13^C magnetic resonance studies. 124. Preparative ring expansions of bicyclic ketones by homoketonization of cyclopropoxide analogs. Can. J. Chem..

[16] Uyehara T., Ohmori K., Kabasawa Y., Kat T. (1984). Alkoxide-accelerated [1,3] Sigmatropic shifts of Bicyclo[3.2.2]non-6-en-2-ols. Substituent effects and tandem [1,3]-[3,3] sigmatropic rearrangements. Chem. Lett..

[17] Lightner D.A., Gawroński J.K., Bouman T.D. (1980). Electronic structure of symmetric homoconjugated dienes. Circular dichroism of (1*S*)-2-deuterio- and 2-methylnorbornadiene and (1*S*)-2-deuterio- and 2-methylbicyclo[2.2.2]octa-diene. J. Am. Chem. Soc..

[18] Fringuelli F., Pizzo F., Taticchi A., Halls T.D.J., Wenkert E. (1982). Diels-Alder reactions of cycloalkenones. 1. Preparation and structure of the adducts. J. Org. Chem..

[19] Northrop B.H., Houk K.N. (2006). Vinylcyclobutane-cyclohexene rearrangement: Theoretical exploration of mechanism and relationship to the Diels-Alder potential surface. J. Org. Chem..

[20] Carpenter B. (1995). Dynamic Matching: The cause of inversion of configuration in the [1,3] sigmatropic migration?. J. Am. Chem. Soc..

[21] Leber P.A., Bell R.M., Christie C.W., Mohrbacher J.A. (2013). A vinylcyclobutane substrate designed as a cyclopropylcarbinyl radical probe. Org. Biomol. Chem..

