# Supplementary material for: Gas Phase Thermal Reactions of exo-8-Cyclopropyl-bicyclo[4.2.0]oct-2-ene (1-exo)
Source: Molecules. 2014 Jan 27;19(2):1527–43. doi: 10.3390/molecules19021527 (PMC6271342; doi:10.3390/molecules19021527)

Supplementary Materials

Table of contents……………………………………………………………………………………S1

Figure S1. ^{1}H and ^{13}C-NMR of *endo*-8-Cyclopropylbicyclo[4.2.0]oct-2-en-6-one (**3**)……………..S2

Figure S2. ^{1}H and ^{13}C-NMR of *exo*-8-Cyclopropylbicyclo[4.2.0]oct-2-ene (**1-*exo***)……………….S3

Figure S3. ^{1}H and ^{13}C-NMR of *endo*-5-Cyclopropylbicyclo[2.2.2]oct-2-ene (**2-*endo***)……………..S4

Figure S4. ^{1}H and ^{13}C-NMR of Bicyclo[3.2.0]non-6-en-2-one (**7**)………………………………….S5

Figure S5. ^{13}C-NMR of Bicyclo[5.2.2]undec-8-en-4-one (**8**)……………………………………….S6

Figure S6. Mass Spectra of Bicyclo[5.2.2]undeca-3,8-diene (***CPC*-1**)…………………………….…...S7

Figure S7. ^{1}H and ^{13}C-NMR of Bicyclo[5.4.0]undec-9-en-2-one (**9**)……….……………………...S8

Figure S8. ^{1}H and ^{13}C-NMR of Bicyclo[5.4.0]undeca-2,9-diene (**10**)………………………………S9

Table S1. Time-dependent Concentration Kinetics for Compound **1-*exo*** @275 ℃.………………S10

Figure S9. Concentration *versus* time plot for thermal reactions of **1-*exo*** @275 ℃.……………...S10

Figure S10. Solver Curve Fit for Compound **1-*exo***.………………………………………………….S11

Figure S11. Solver Curve Fit for Compound **1-*endo***.………………………………………………...S11

Figure S12. Solver Curve Fit for Compound ***CPC*-1**.………………………………………………...S12

Figure S13. Concentration *versus* time plot for [1,3] thermal products (**2-*exo*** and ***2-endo***).……...S12

Figure S14. Concentration *versus* time plot for fragmentation, both direct and indirect.………….S13

Figure S1. ^{1}H and ^{13}C-NMR of *endo*-8-Cyclopropylbicyclo[4.2.0]oct-2-en-6-one (**3**).

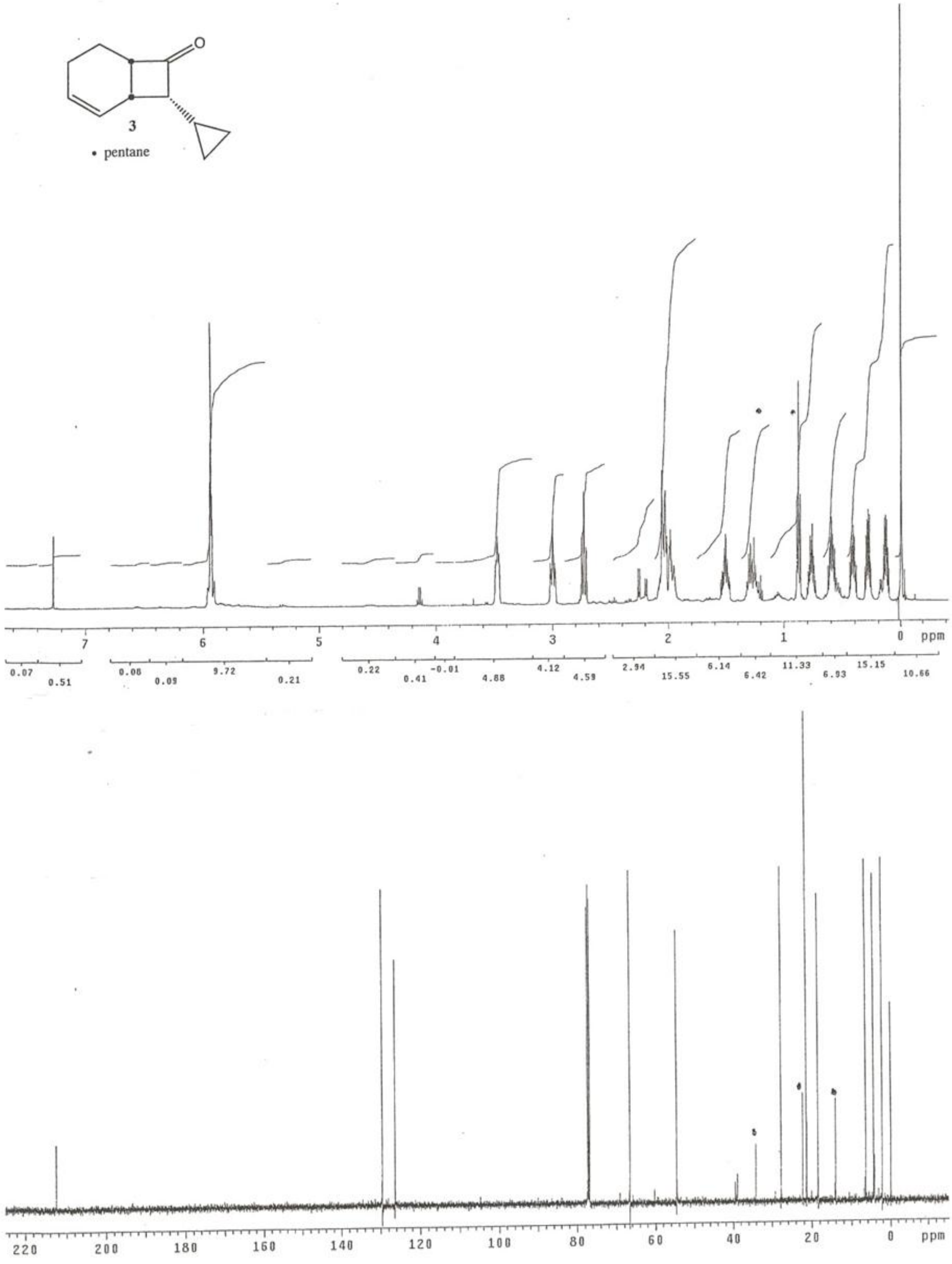

Figure S2. ^{1}H and ^{13}C-NMR of *exo*-8-Cyclopropylbicyclo[4.2.0]oct-2-ene (**1-*exo***).

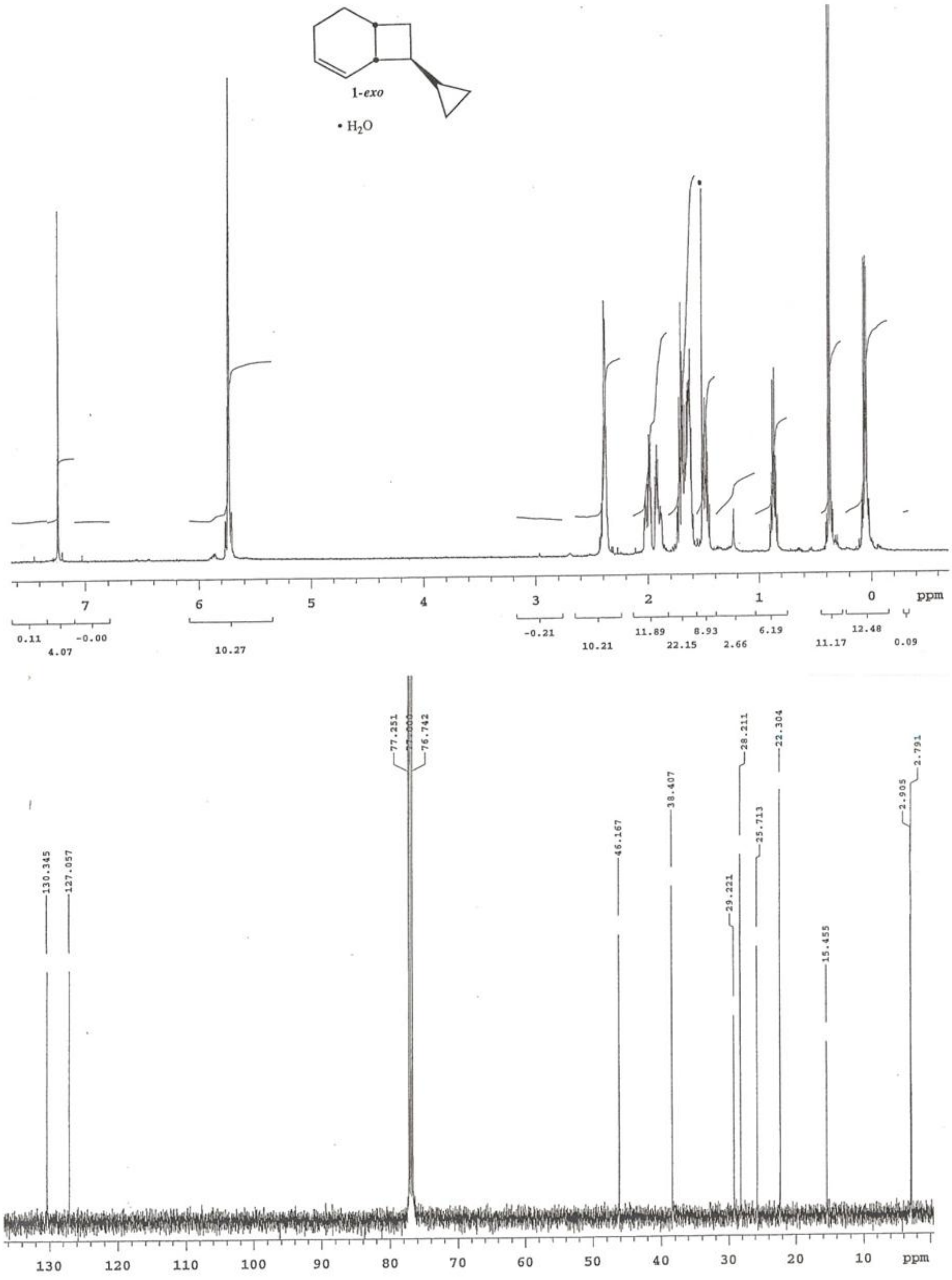

Figure S3. ^{1}H and ^{13}C-NMR of *endo*-5-Cyclopropylbicyclo[2.2.2]oct-2-ene (**2-*endo***).

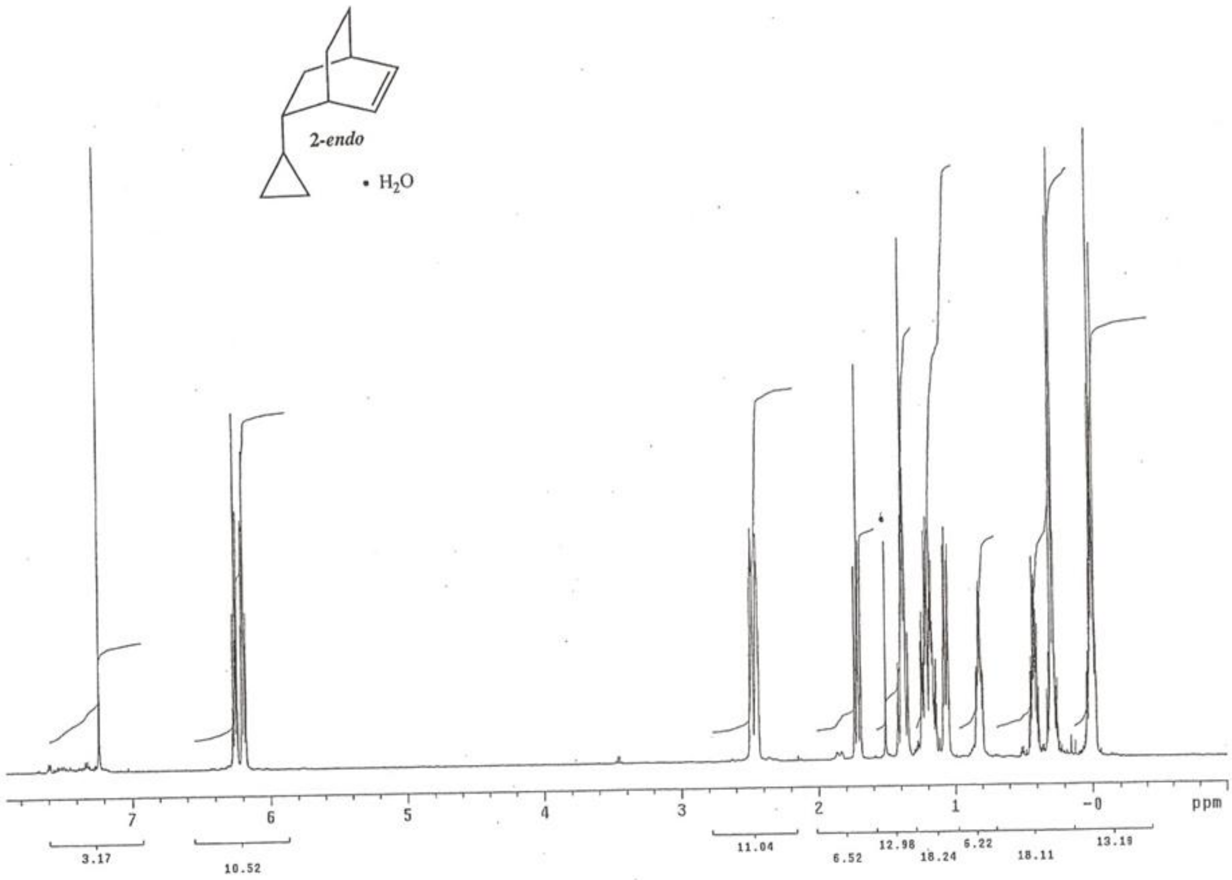

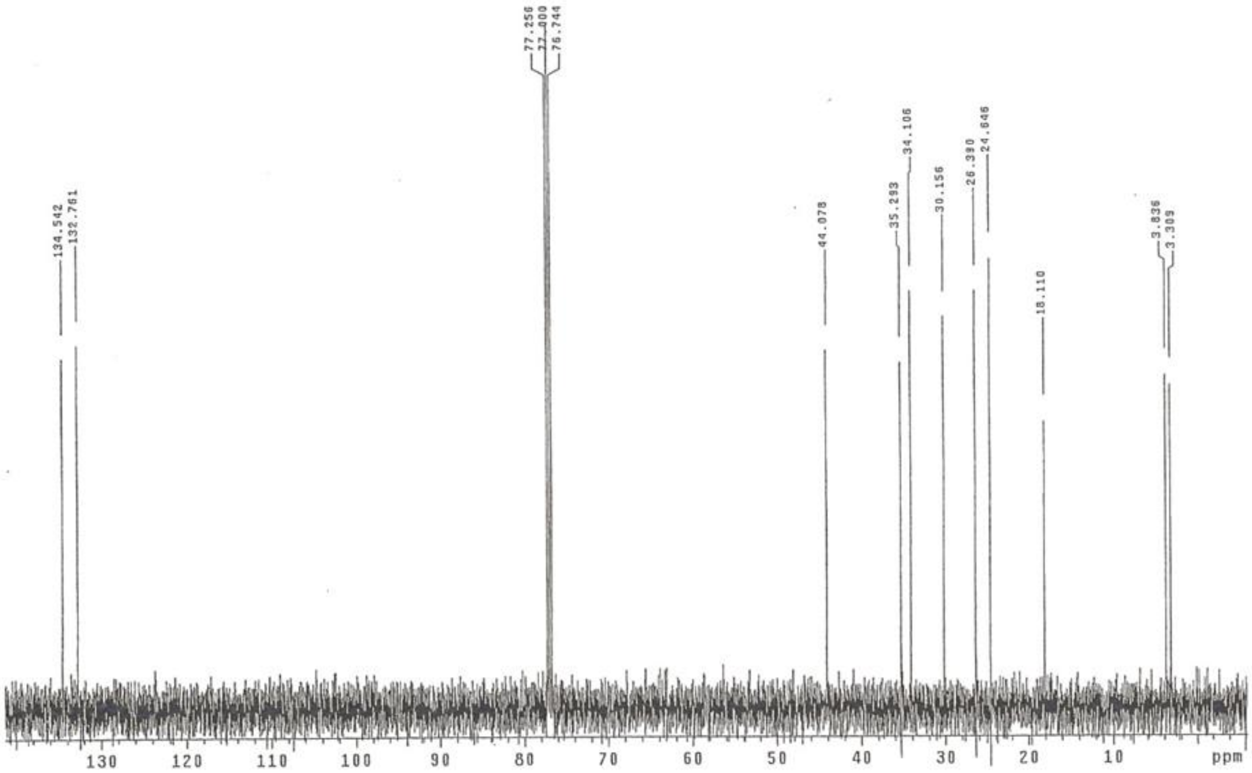

Figure S4. ^{1}H and ^{13}C-NMR of Bicyclo[3.2.0]non-6-en-2-one (**7**).

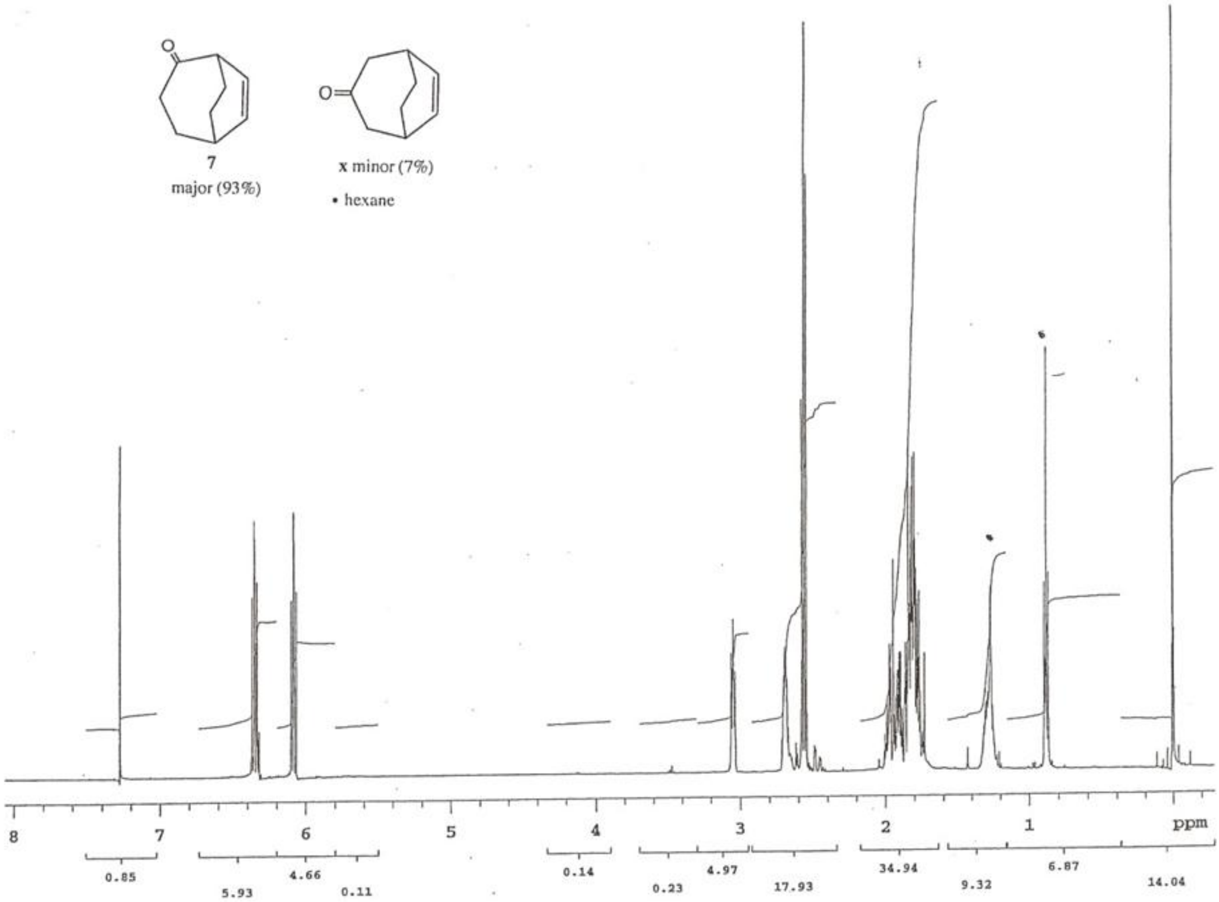

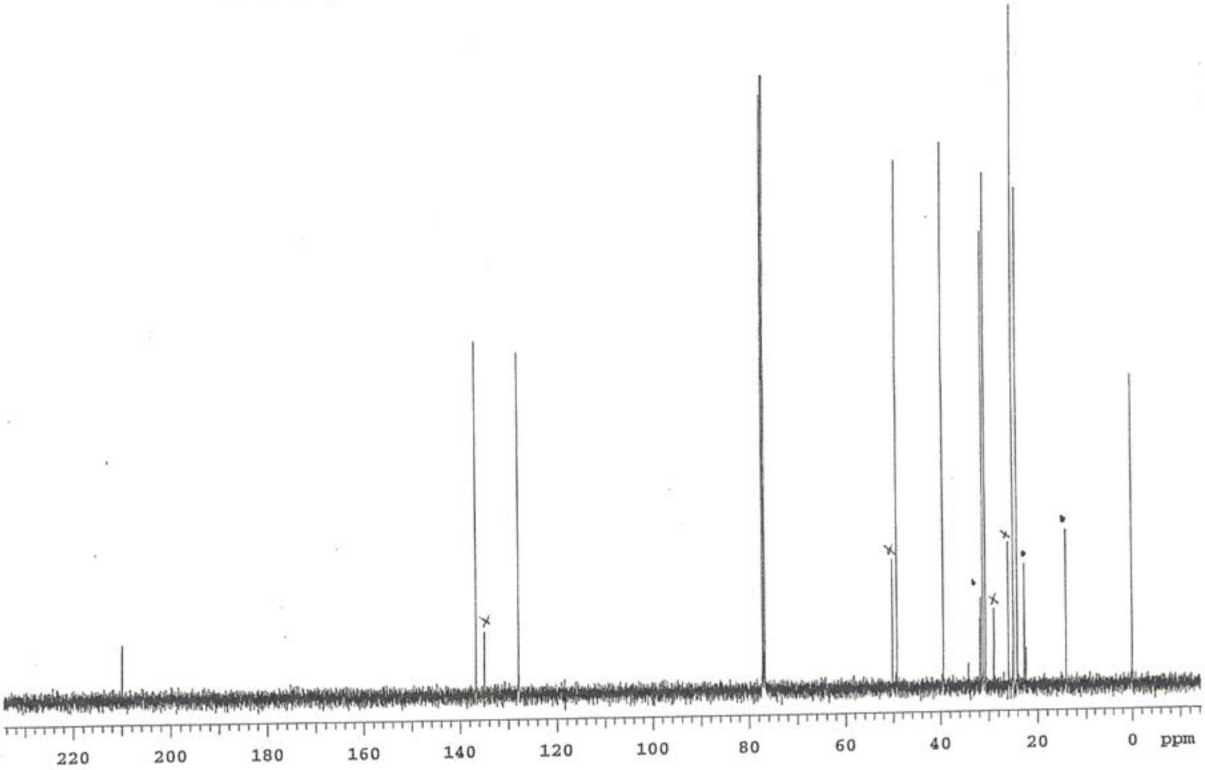

Figure S5. ^{13}C-NMR of Bicyclo[5.2.2]undec-8-en-4-one (**8**).

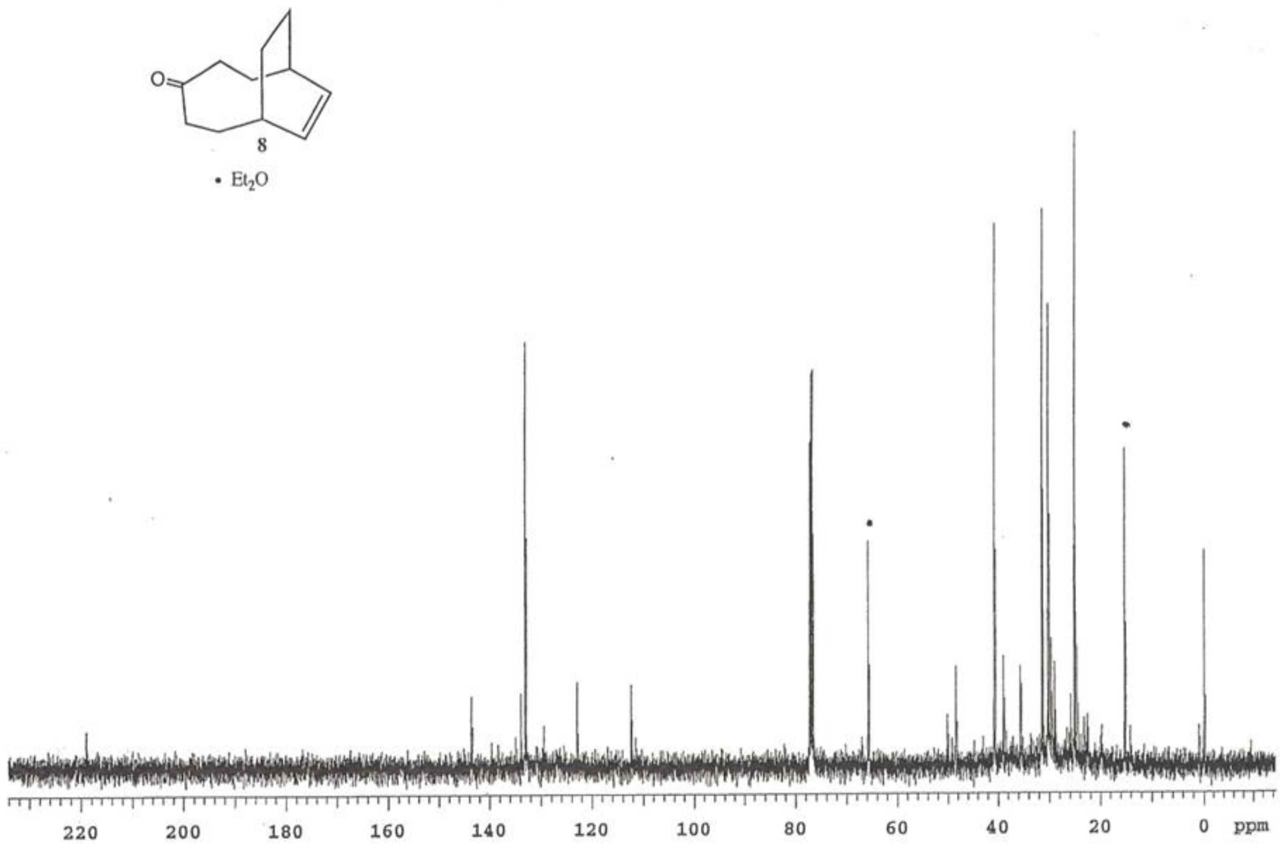

Figure S6. Mass Spectra of Bicyclo[5.2.2]undeca-3,8-diene (***CPC*-1**).

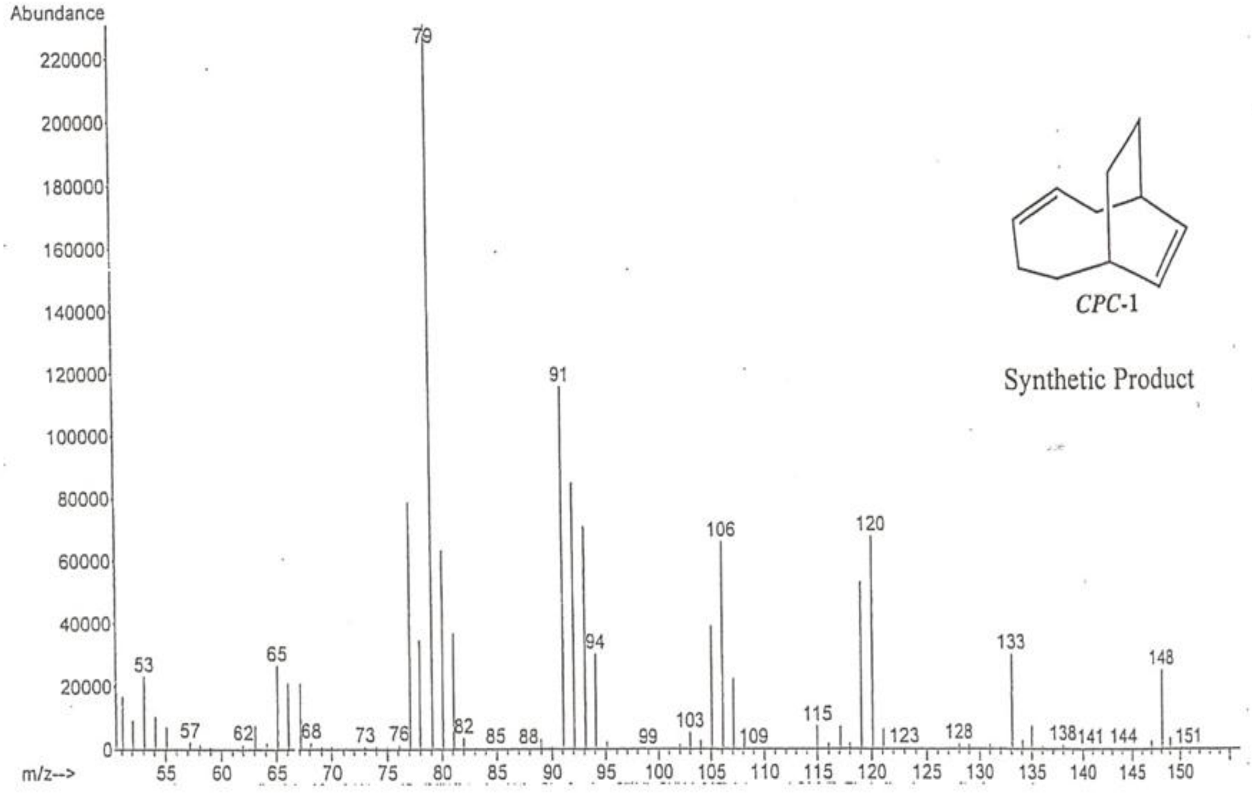

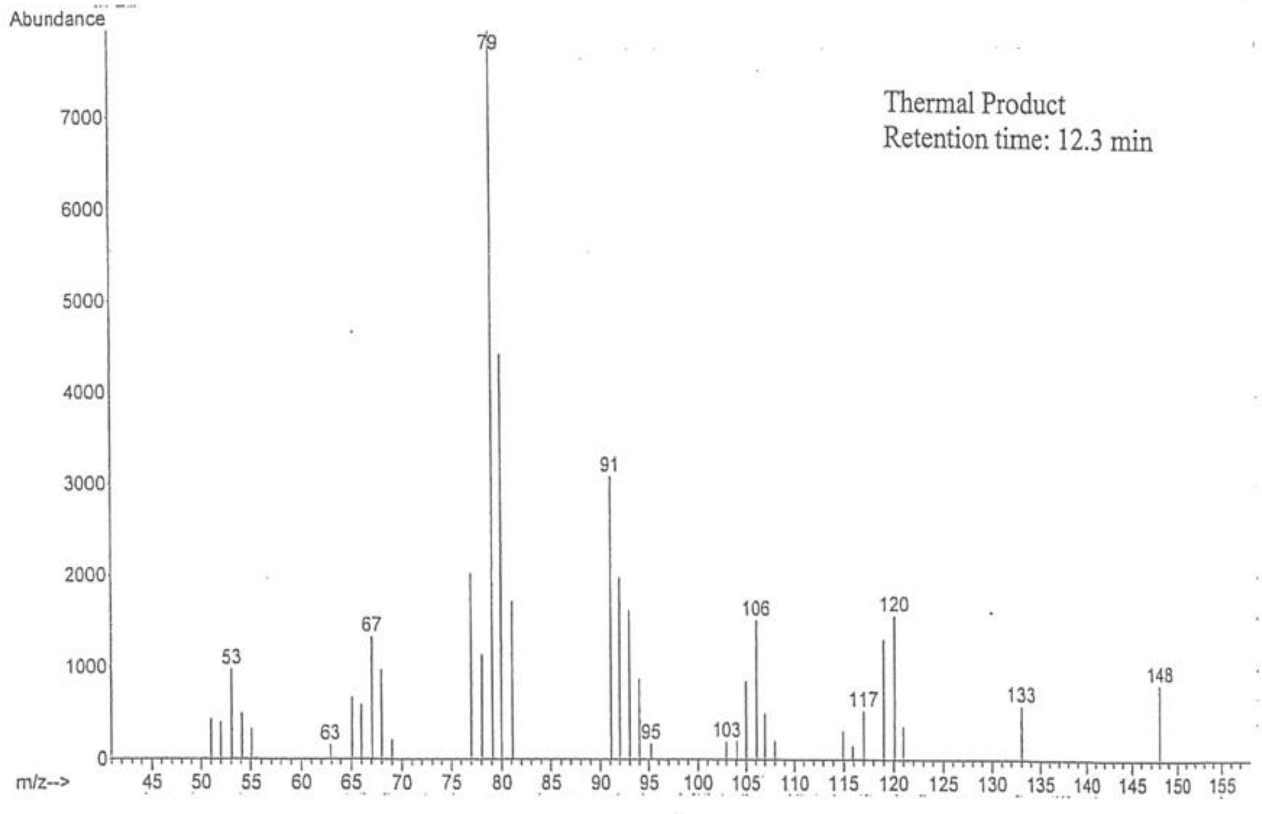

Figure S7. ^{1}H and ^{13}C-NMR of Bicyclo[5.4.0]undec-9-en-2-one (**9**).

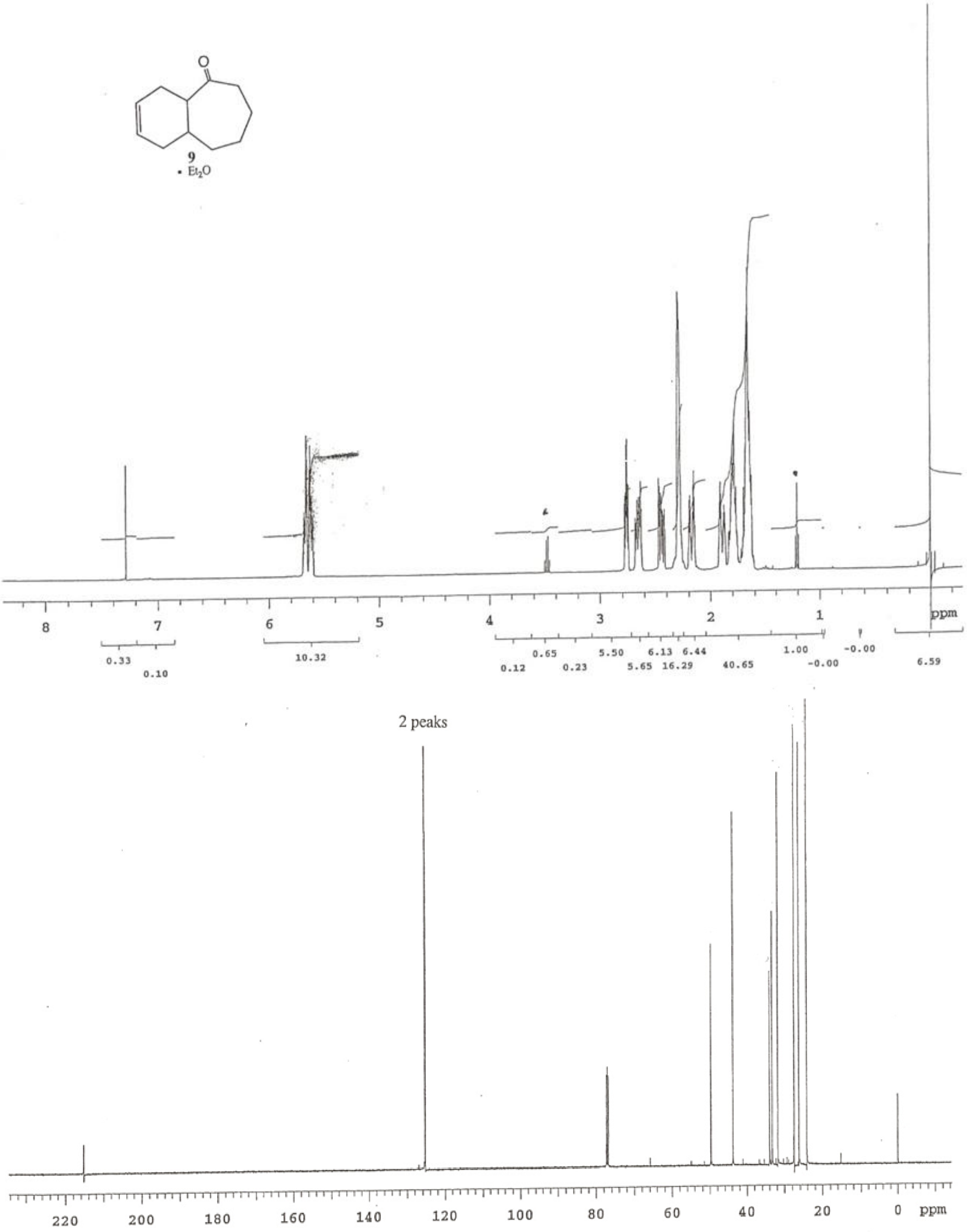

Figure S8. ^{1}H and ^{13}C-NMR of Bicyclo[5.4.0]undeca-2,9-diene (**10**).

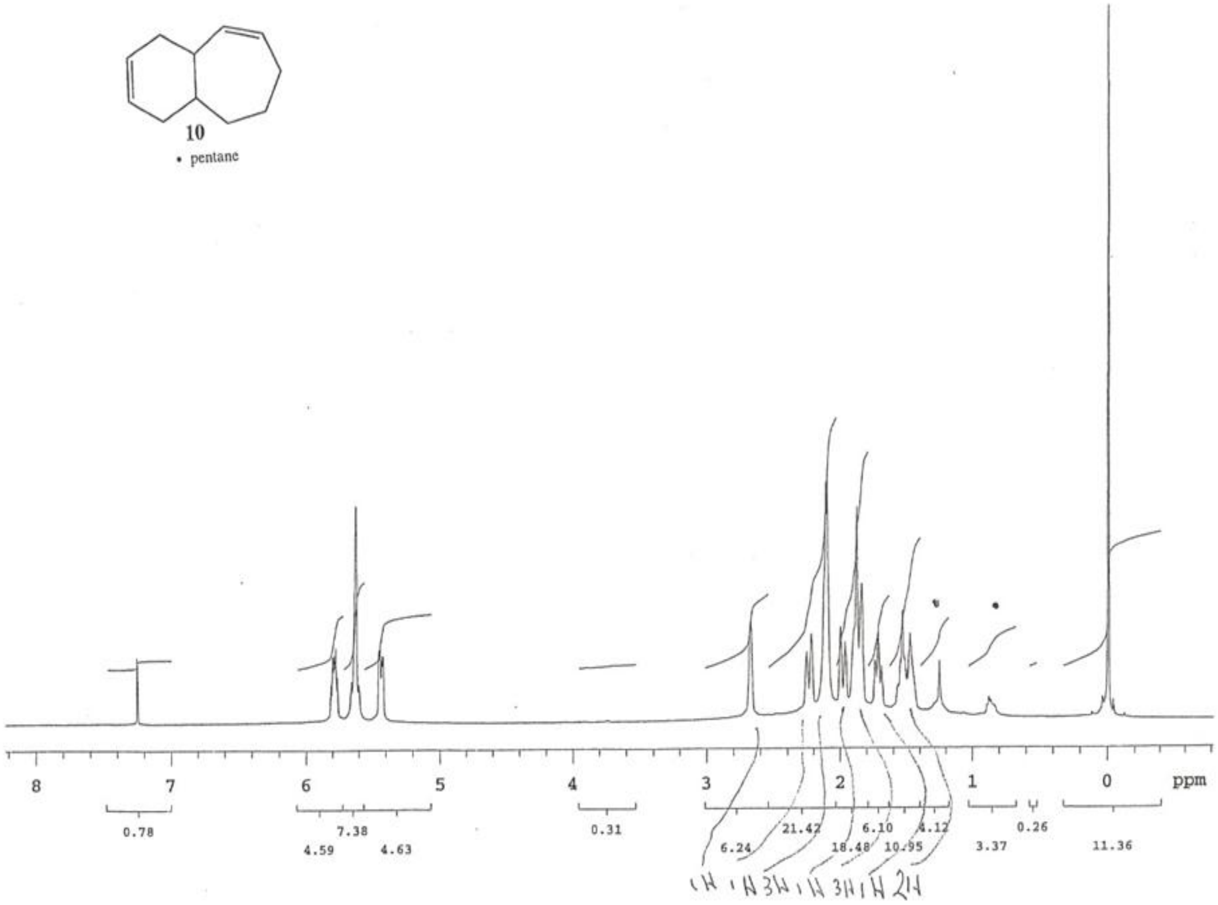

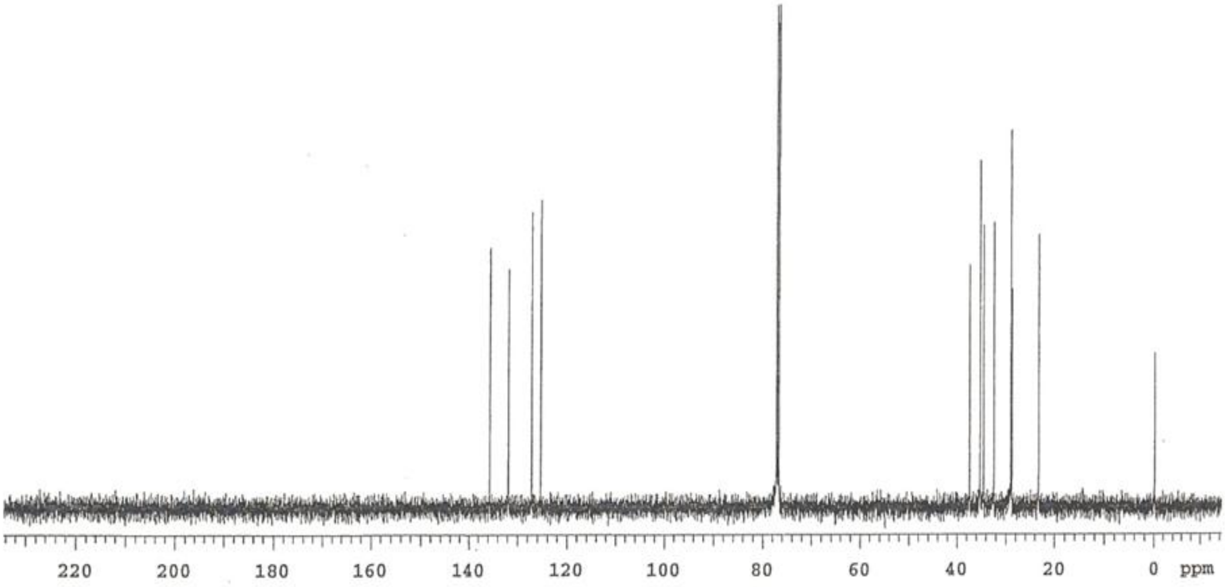

Table S1. Time-dependent Concentration Kinetics for Compound **1-*exo*** @275 ℃.

Time (h)	**2-*endo*** (*sr*)	**2-*exo*** (*si*)	***CPC*-1**	**1-*exo***	**1-*endo***	fragments
0.0	0	0	0	1.00000	0	0
1.0	0.00953	0.01654	0*	0.86826	0.06619	0.03948
2.0	0.01750	0.03105	0.00335	0.76518	0.10194	0.08098
4.0	0.02805	0.05152	0.00622	0.57212	0.12505	0.21704
6.0	0.03942	0.07193	0.00930	0.47742	0.13000	0.27193
7.9	0.05084	0.09323	0.01161	0.42208	0.12870	0.29354
10.0	0.05955	0.10824	0.01444	0.34375	0.11278	0.36123
15.8	0.07450	0.13734	0.01745	0.21397	0.07333	0.48340
23.3	0.08815	0.15824	0.01898	0.11277	0.03944	0.58240
35.4	0.10271	0.17862	0.02137	0.05111	0.01719	0.62898

* Value fell below integration threshold on the analytical GC recorder/integrator.

Figure S9. Concentration *versus* time plot for thermal reactions of **1-*exo*** @275 ℃.

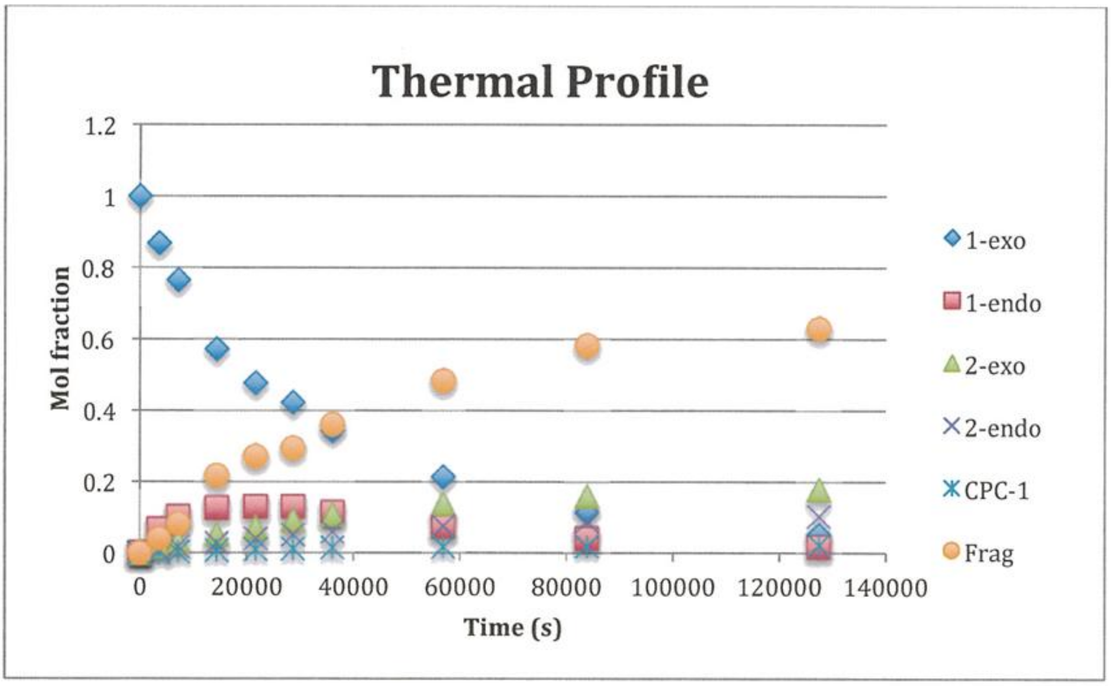

Figure S10. Solver Curve Fit for Compound **1-*exo***.

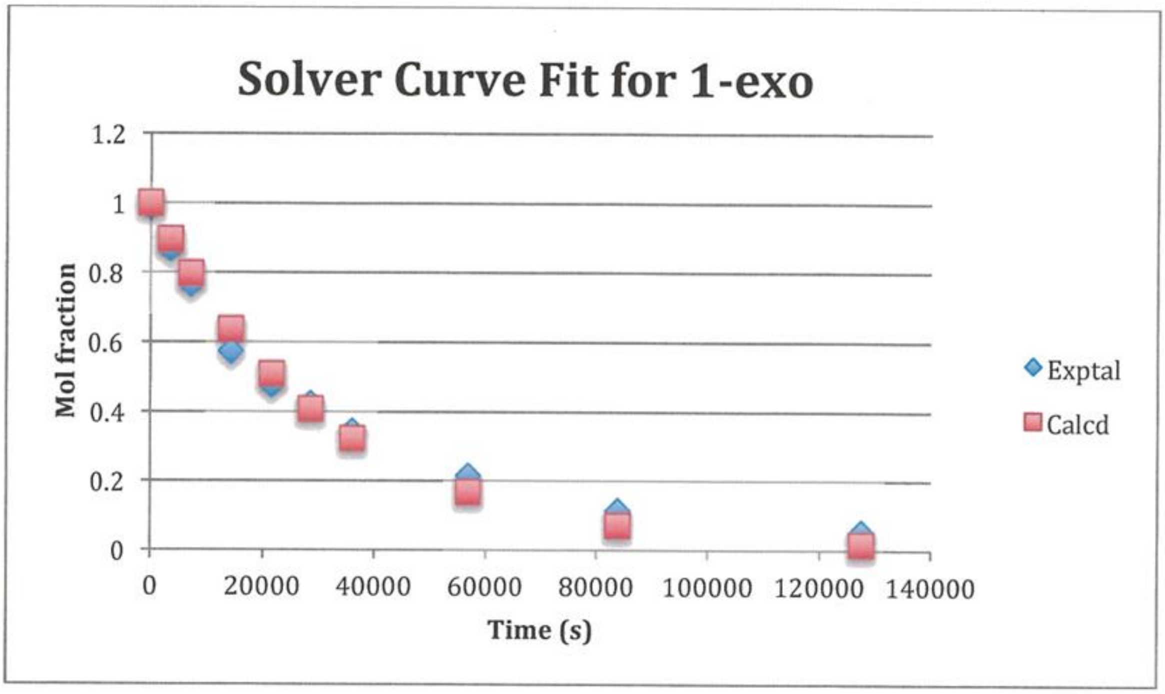

Figure S11. Solver Curve Fit for Compound **1-*endo***.

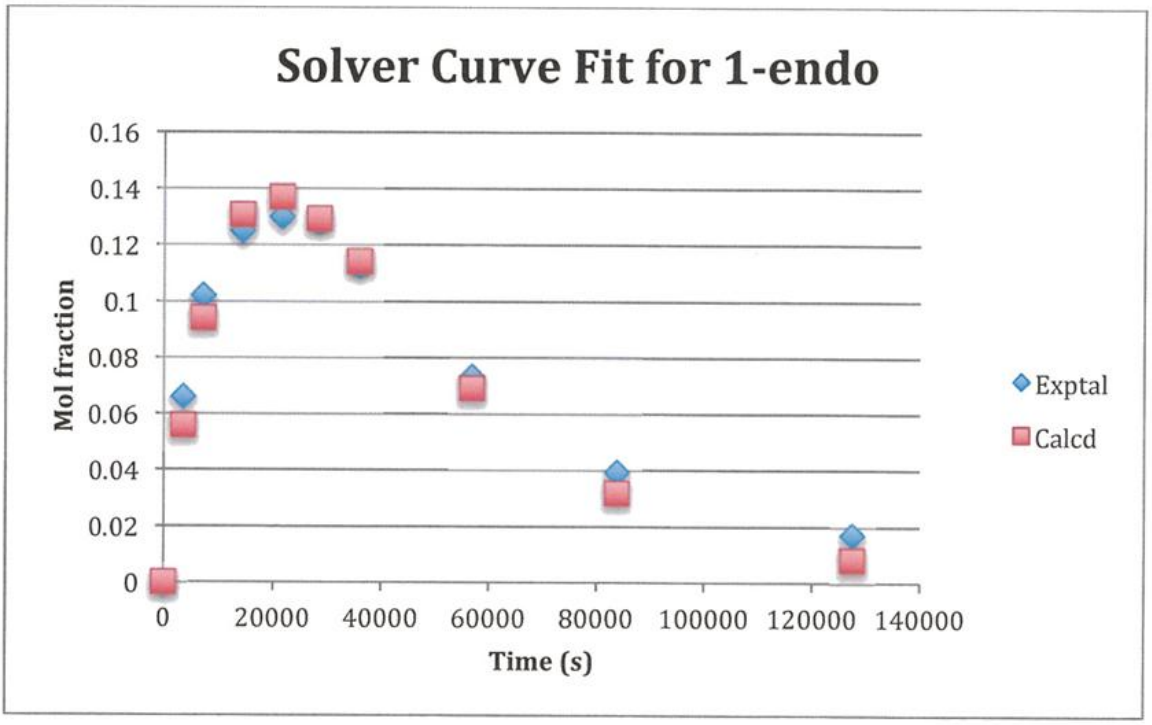

Figure S12. Solver Curve Fit for Compound ***CPC*-1**.

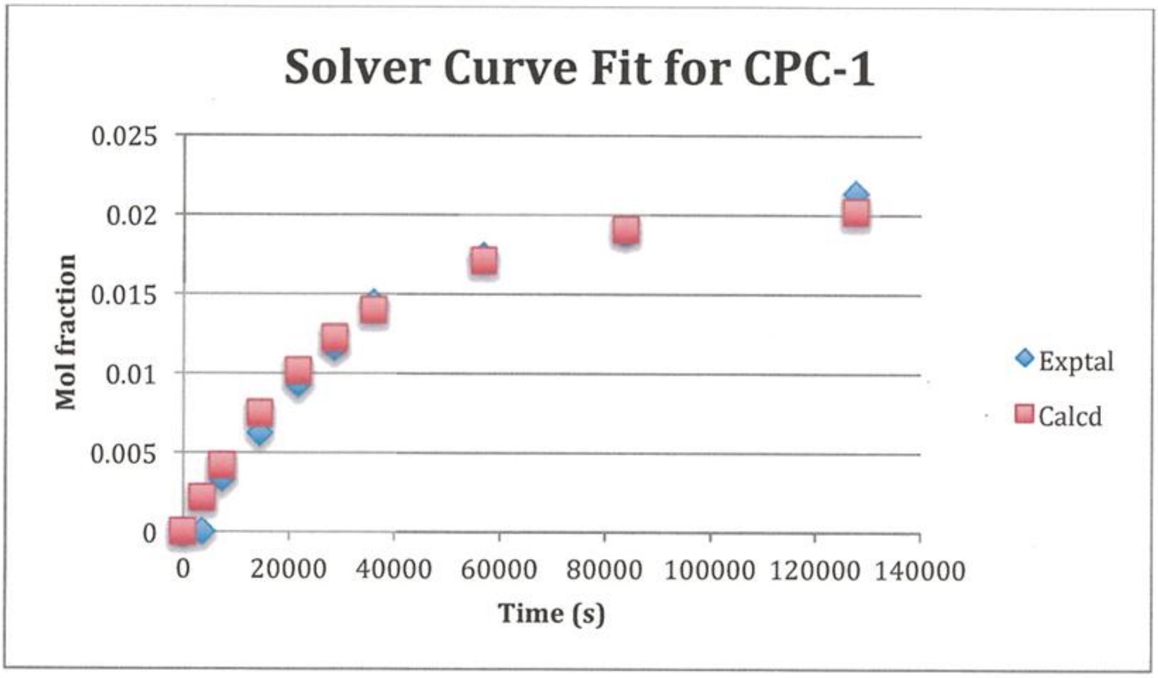

Figure S13. Concentration *versus* time plot for [1,3] thermal products (**2-*exo*** and ***2-endo***).

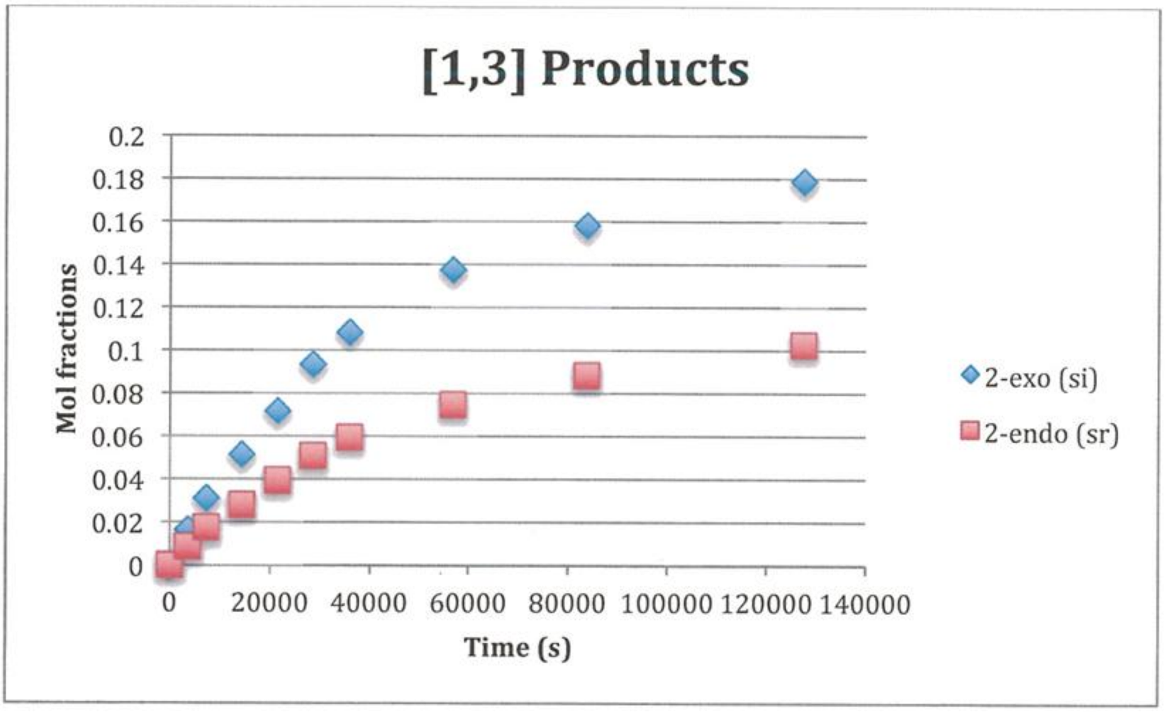

Figure S14. Concentration *versus* time plot for fragmentation, both direct and indirect.

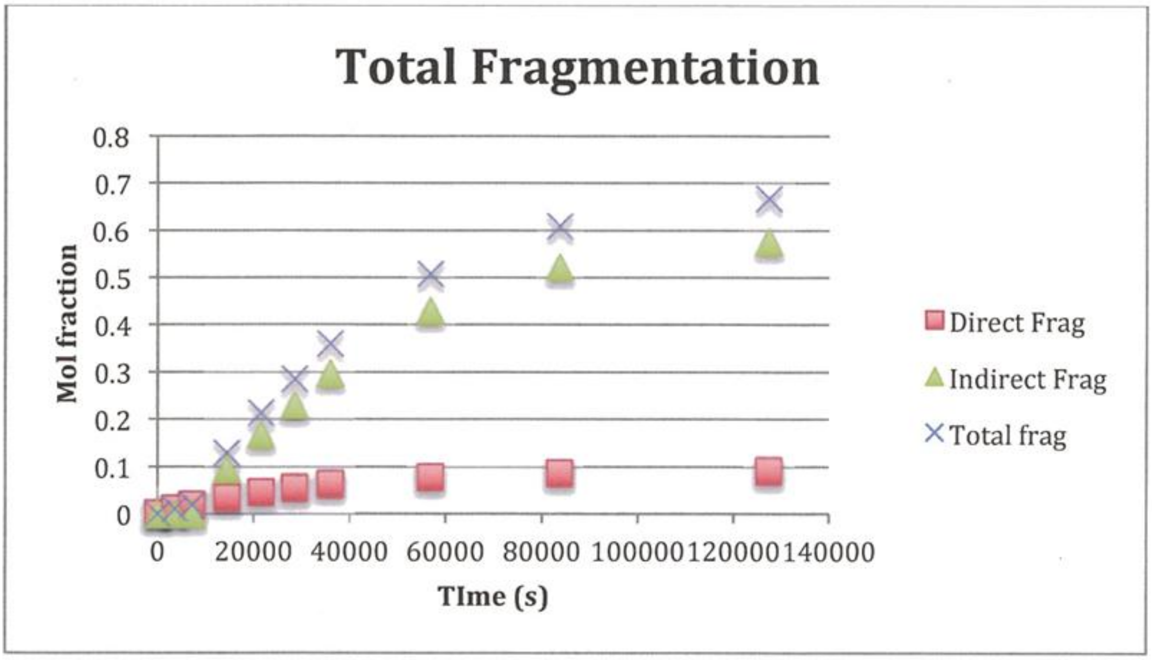

Supplement: Supplementary file 1 [file molecules-19-01527-s001.pdf]
